# How drugs get into cells: tested and testable predictions to help discriminate between transporter-mediated uptake and lipoidal bilayer diffusion

**DOI:** 10.3389/fphar.2014.00231

**Published:** 2014-10-31

**Authors:** Douglas B. Kell, Stephen G. Oliver

**Affiliations:** ^1^School of Chemistry, The University of ManchesterManchester, UK; ^2^Manchester Institute of Biotechnology, The University of ManchesterManchester, UK; ^3^Department of Biochemistry, University of CambridgeCambridge, UK; ^4^Cambridge Systems Biology Centre, University of CambridgeCambridge, UK

**Keywords:** drug transporters, systems pharmacology, pharmacogenomics, Recon2

## Abstract

One approach to experimental science involves creating hypotheses, then testing them by varying one or more *independent* variables, and assessing the effects of this variation on the processes of interest. We use this strategy to compare the intellectual status and available evidence for two models or views of mechanisms of transmembrane drug transport into intact biological cells. One (BDII) asserts that lipoidal phospholipid Bilayer Diffusion Is Important, while a second (PBIN) proposes that in normal intact cells Phospholipid Bilayer diffusion Is Negligible (i.e., may be neglected quantitatively), because evolution selected against it, and with transmembrane drug transport being effected by genetically encoded proteinaceous carriers or pores, whose “natural” biological roles, and substrates are based in intermediary metabolism. Despite a recent review elsewhere, we can find no evidence able to support BDII as we can find no experiments in intact cells in which *phospholipid bilayer* diffusion was either varied independently or measured directly (although there are many papers where it was inferred by seeing a *covariation* of other *dependent* variables). By contrast, we find an abundance of evidence showing cases in which changes in the activities of named and genetically identified transporters led to measurable changes in the rate or extent of drug uptake. PBIN also has considerable predictive power, and accounts readily for the large differences in drug uptake between tissues, cells and species, in accounting for the metabolite-likeness of marketed drugs, in pharmacogenomics, and in providing a straightforward explanation for the late-stage appearance of toxicity and of lack of efficacy during drug discovery programmes despite macroscopically adequate pharmacokinetics. Consequently, the view that Phospholipid Bilayer diffusion Is Negligible (PBIN) provides a starting hypothesis for assessing cellular drug uptake that is much better supported by the available evidence, and is both more productive and more predictive.

## Introduction

“The overthrow of the phlogiston theory involved the development of a superior conceptual theme” (Conant, 1950).

As part of an ongoing discussion of the importance of transporters in drug distribution that we (Dobson and Kell, 2008; Dobson et al., 2009a,b; Kell and Dobson, 2009; Kell et al., 2011, 2013; Lanthaler et al., 2011; Kell, 2013; Kell and Goodacre, 2014) and others (e.g., Sai and Tsuji, 2004; Shitara et al., 2006; Anderson and Thwaites, 2010; Franke et al., 2010; Giacomini et al., 2010, 2013; Lai et al., 2010; Burckhardt and Burckhardt, 2011; Fromm and Kim, 2011; König, 2011; Mruk et al., 2011; Nies et al., 2011; Thompson, 2011; Tirona, 2011; Zolk and Fromm, 2011; Degorter et al., 2012; Mandery et al., 2012; Riedmaier et al., 2012; Sprowl et al., 2012; Chu et al., 2013b; Estudante et al., 2013; Giacomini and Huang, 2013; Hagenbuch and Stieger, 2013; König et al., 2013; Schlessinger et al., 2013a,b; Tamai and Nakanishi, 2013; Lai and Hsiao, 2014; Sprowl and Sparreboom, 2014) have been highlighting, Smith and colleagues recently published a review (Smith et al., 2014) that claims that the hypothesis that drugs are usually transported into cells via protein carriers is “not a sound scientific principle and lacks experimental evidence.” Smith et al. (2014) set out their arguments in considerable detail, and this allows us, in the present publication, to present a contrary view and rehearse the core arguments that pertain to the mechanism(s) of drug and xenobiotic transport across biological membranes.

**Figure 1:**
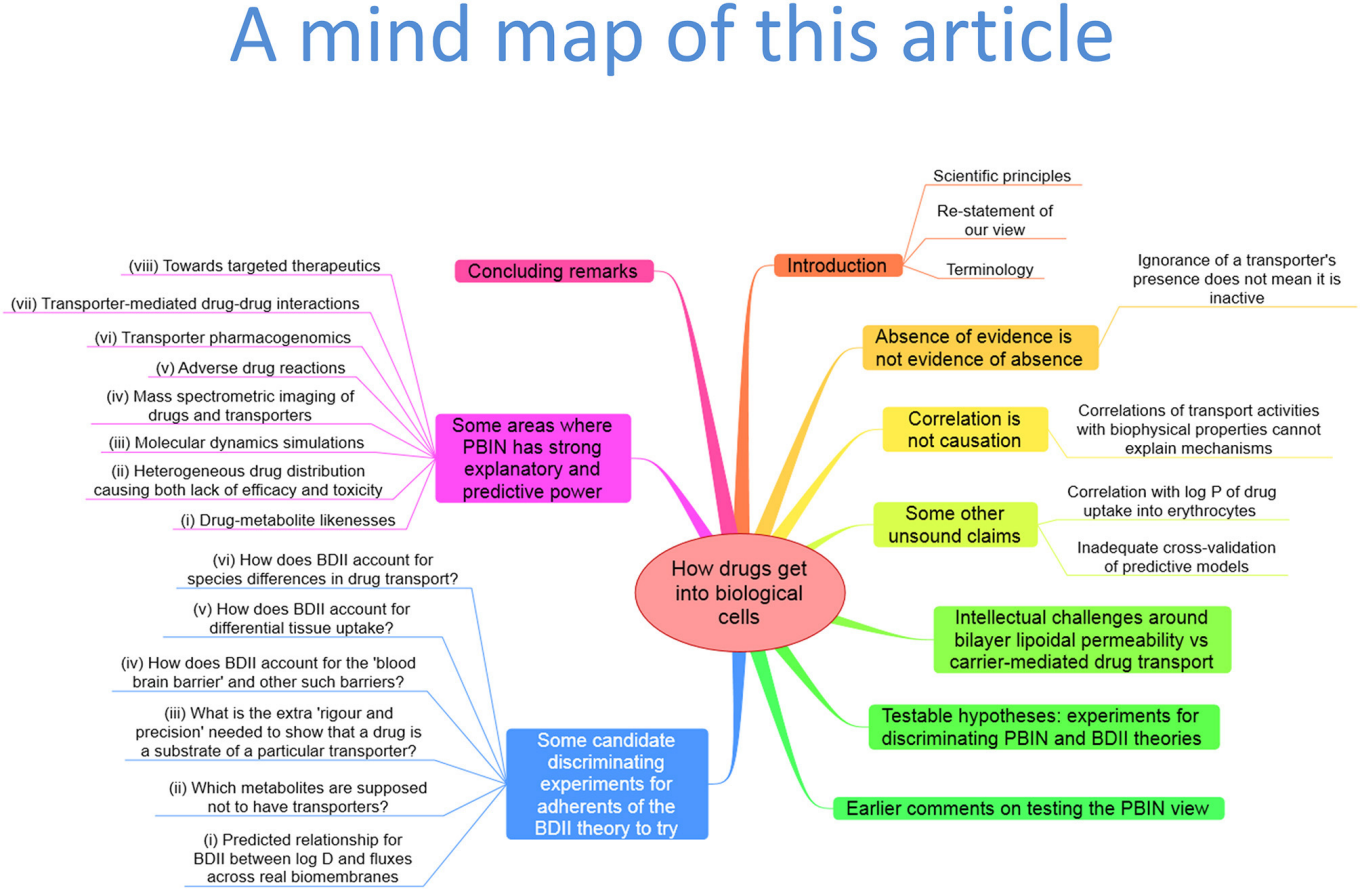
**A “mind map” (Buzan, 2002) summarizing the structure and contents of this paper**. To follow this, start at the top and read clockwise.

First, we might usefully establish (or, more accurately, restate) what our views are. The abstract of the Smith article (Smith et al., 2014) (“Recently, it has been proposed that drug permeation is essentially carrier-mediated only and that passive lipoidal diffusion is negligible”) recognizes that we imply a *dominant* role for transporter-mediated uptake of drugs into cells (note the titles of Dobson and Kell, 2008; Dobson et al., 2009a; Kell and Dobson, 2009; Kell et al., 2011, 2013). We do not assert that carrier-mediated transport is the *only* means by which drugs and other xenobiotics gain access to cells, nor do we seek to invalidate passive lipoidal diffusion as an alternate mechanism. Thus, we start by explaining, from a Popperian standpoint, why we do not seek to “invalidate” bilayer lipoidal diffusion. Figure 1 provides an overview of this article in the form of a mind map (Buzan, 2002).

### Scientific principles

A well-known scientific principle is that of hypothesis-driven or hypothetico-deductive science and scientific reasoning. It is due in its most widely recognized form to Karl Popper [see (Medawar, 1982; Popper, 1992; Chalmers, 1999), and for its iterative contrast with data-driven approaches see (Kell and Oliver, 2004; Franklin, 2005; Kell, 2006, 2012; Elliott, 2012)]. In this view (as it is applied to experimental science), one produces a hypothesis that allows one to vary something as an independent variable (properly, a parameter), and predicts the observable effects (data) to which one's hypothesis would lead, within a deductive framework. The data observed are then consistent or otherwise with those predicted on the basis of the hypothesis. In the Popperian view, then, hypotheses are there to be refuted but cannot be “proven.” So, while we consider that bilayer lipoidal diffusion is normally probably negligible (i.e., may be neglected in quantitative terms) in intact biological cells, at no time have we tried to “invalidate” passive lipoidal diffusion across real and intact biological membranes, because we have neither tried to measure it directly nor to vary it as an independent variable. Neither, so far as we can tell, has anyone else. Thus, we merely point out that there is no actual *evidence* for it occurring in normal biomembranes; what there are (in abundance) are *data sets* of e.g., drugs appearing in cells when added externally, and we note that people choose to *interpret* this as evidence somehow supporting bilayer diffusion, but that is not at all the same thing (Ioannidis, 2005; Broadhurst and Kell, 2006) and, in fact, direct experimentation suggests quite the opposite.

**Figure 2:**
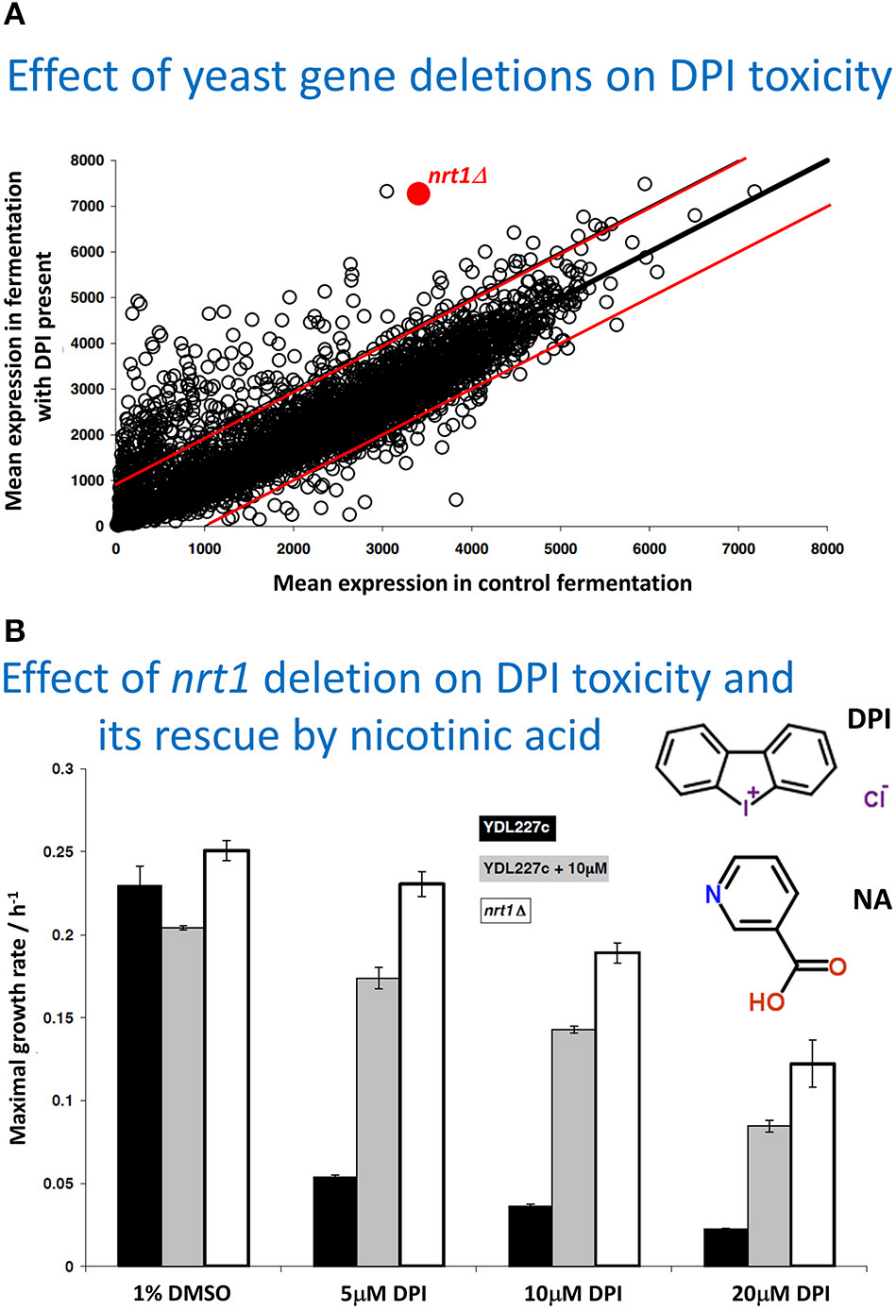
**Variation of transporter expression in the yeast gene knockout collection, as an independent variable, leads to measurable changes in the selection of different strains when exposed to toxic concentrations of a drug (here diphenyleneiodonium chloride, DPI), shown in Figure (A)**. The experiment is replotted and reannotated from Lanthaler et al. (2011). **(A)** the strains are competed in a fermentor that either does or does not contain diphenyleneiodonium, each strain being an independent variable, and their effective selection plotted as the mean amount of each strain (on the ordinate) remaining relative to the mean amount of each strain in the controls (on the abscissa), both being dependent variables. The data imply that resistance to the drug is conferred when the nrt1 transporter is knocked out, and that this is the ones to test directly for transporter activity. The two red lines show the range in which 98% of deletant lie in duplicate control experiments, thereby giving an indication of the experimental noise. **(B)**. Assessment via maximal growth rate of the resistance of wild type (YDL 227c) and *nrt1* deletant strains to different amounts of DPI and its protection by 10 μM nicotinic acid (NA). The structures of DPI and NA are also shown (NA was used as nicotinamide riboside was unavailable). For further information, see Lanthaler et al. (2011).

So, to be clear: our views are that we find no serious *evidence* for bilayer lipoidal diffusion of drugs into cells. A major reason for our thinking comes from the fact there are so many cases (that we discuss below) in which drugs or other natural and xenobiotic molecules simply do not seem to enter or exit from cells, at least without identifiable transporters being present. This implies that the “background” rate of transport (from the exterior all the way into the aqueous cytoplasm) through any bilayer, as assumed to be present in all mammalian cells, must be negligible. We also consider it likely that evolution long ago selected against cells that might not be osmotically active if they became permeable to all kinds of small molecules. By contrast, we find much evidence (almost wherever we look) for the presence of carrier-mediated transport (whatever interpretations may be put on the *data* in any specific papers, whether by their original authors or by commentators), because such molecules allow for controlled permeability, and transporter activities can be and are varied experimentally *as independent variables with predictable and measurable effects*. Figure 2 re-plots data from a competition experiment (Lanthaler et al., 2011) in which we compared the ability of baker's yeast strains carrying single-gene deletants to display resistance to a toxic concentration of a drug (in this case diphenyleneiodonium, DPI), the idea being that a strain lacking a non-essential transporter for the drug would display resistance. We would also stress that this experiment can only work effectively to discriminate between the different mutant strains when any *background* Phospholipid Bilayer diffusion Is Negligible (PBIN). If there was a significant transporter-independent background rate, all strains would be selected (or otherwise) to virtually the same extent. Figure 2A highlights one transporter that displays a significant extent of such resistance (as the *nrt1* deletant). *NRT1* encodes a nicotinamide riboside transporter, which led us to hypothesize (i) that such a deletant would display resistance to DPI when cultured axenically, and (ii) that nicotinic acid would be able to compete with the DPI and effect phenotypic resistance. Figure 2B shows that both predictions are entirely fulfilled.

A related aspect of the hypothesis-driven approach, and note that we do also recognize the great (and perhaps greater) value of data-driven approaches (Kell and Oliver, 2004), is the view that a good theory or hypothesis has predictive power both for existing data and for other experiments not yet done. We give examples later.

### Re-statement of our view

Our view for the transport of xenobiotic molecules (that include drugs) into intact cells might better be referred to as being that, in normal intact cells, drug molecules do not mysteriously float across any untrammeled bilayer portions of membranes that may exist, and thus that PBIN. This does not therefore have anything to say about endocytosis, paracellular transport or other modes of drug passage within tissues. Various *corollaries* or *contingent and testable hypotheses* follow from this (and see later), however—for instance that in many cases one ought to be able to find the transporters, that the permeability to drugs of intact biological membranes that lack any suitable transporters is negligible, and that transporter-mediation can easily account for the very heterogeneous distributions of drugs between different cells, tissues, individuals, or species. The PBIN hypothesis has the benefit of being simple (as per Occam's razor Westerhoff et al., 2009) and has high explanatory power for phenomena that we think are otherwise hard to explain on an alternative hypothesis that phospholipid Bilayer Diffusion Is Important (BDII). The Occam's razor argument means that if we can explain available data in terms of transporters (PBIN vs. BDII) without any need to invoke bilayer diffusion, then PBIN is a preferable hypothesis, that like all good hypotheses can also be tested with well-designed (i.e., genuinely discriminating) experiments and, in principle, refuted in particular cases.

### Terminology

Let us also restate and clarify the confused terminology that dogs this field: (i) “active” transport means uptake or indeed efflux (usually—and in this context more or less inevitably—via one or more transporters) that is concentrative in nature (and necessarily driven by an “external” energy source); (ii) “passive” transport simply means transport that is not concentrative in nature, i.e., it is equilibrative of transmembrane thermodynamic activities. Often, the latter is *assumed* to mean via lipoidal diffusion, but in general (in the field of biological transmembrane transport) no mechanism is implied by the term, and it is best if one always states what (if any) mechanism is implied. If we state “bilayer lipoidal” that is what we mean, but if we do not state a mechanism we leave it open. Passive transport *through transporters* is also equilibrative (of thermodynamic activities, related to free concentrations) and is normally (and correctly and usefully) referred to as “facilitated diffusion.”

## Absence of evidence is not evidence of absence: if you do not know about a drug transporter, it does not mean that it is not there and active

A typical device used by those claiming “evidence” for the BDII hypothesis is to find a system and substrate in which there is uptake that is at least partially through a *known* transporter, to inhibit that transporter, and then simply to state that the rest of the uptake is *therefore* by lipoidal diffusion. This is, rather obviously, an inadequate and illogical interpretation since, in most cases, where one transporter is known so are a number of others [e.g., (Kell et al., 2011, 2013; Lanthaler et al., 2011; Sprowl and Sparreboom, 2014), and the same is true for ligands generally (Kell et al., 2013)]. At all events, it is clearly illogical to consider that this constitutes any kind of evidence for BDII when one knows nothing about the other transporters that may be active in the same tissues on the substrate of interest (Kell et al., 2011, 2013).

Quoting from a recent example Smith et al. (2014): “Additional recent (*sic*) data (Xu et al., 1998) from fluorine NMR studies on uptake of modified nucleosides (L-FMAU) into erythrocytes (biological systems that include transporters) provide clear indication (*sic*) of two different mechanism (*sic*) governing uptake of L-FMAU in erythrocytes: facilitated transport via nucleoside transporter and non-facilitated diffusion.” In fact, they do not. What Xu et al. (1998) actually showed was that some of the uptake of the nucleoside was inhibited by an inosine analog, considered to be an inhibitor of “the” nucleoside transporter, but the rest of the uptake was not affected either by thiol reagents or by uracil (a substrate for a nucleobase—rather than nucleoside—transporter). They did not actually *measure* lipoidal diffusion—they simply assumed it on the basis that they were not aware of any other transporters, so there is not even the possibility of a “clear indication.” However, in the 16 years since that publication, what we do have clear evidence for is that there are at least *seven* major transporters for nucleosides in humans (He et al., 2009; Hediger et al., 2013) (http://slc.bioparadigms.org/) [*viz*. concentrative nucleoside transporters CNT1-3 of the SLC (Schlessinger et al., 2013b) 28 family (Gray et al., 2004; Young et al., 2013) and equilibrative nucleoside transporters ENT1-4 of the SLC29 family (Baldwin et al., 2004; Young et al., 2013)], albeit some nucleobases will also use these transporters (Quashie et al., 2008, 2010). Of the SLC29 family, mainly ENT1 seems to be expressed in erythrocytes (Endres et al., 2009a,b), but there are indications that other transporters contribute to the very active nucleoside uptake into erythrocytes (Löffler et al., 2007); the expression levels in erythrocytes of the other SLC29 family members and of the widely expressed CNT1-3 are apparently unknown. All nucleoside transporters (including those of the CNT family) are expressed, often quite strongly, in lymphocytes (Conklin et al., 2012), however. There are also some 31 nucleotide-sugar transporters (members of the SLC35 family; Ishida and Kawakita, 2004; Song, 2013) whose expression levels and specificities in erythrocytes are not known, and other transporters may also be involved (Trigueros-Motos et al., 2012). In conclusion, it is not appropriate to claim “evidence” for a process (lipoidal diffusion) when one is not in fact measuring it, but simply assuming or inferring it (while ignoring many other possible mechanisms).

## Correlation of particular activities with any other activities or with biophysical properties is not causation and cannot explain mechanisms

Many papers used to construct arguments as to the mechanisms of transmembrane drug transport show correlations between various things and take them as evidence for mechanisms (particularly BDII). We have pointed out many times that these kinds of correlations show nothing except that they exist in the systems stated. An attempt to indicate causation requires that something is varied (and usually plotted on the abscissa) *as an independent variable*. Note that dependent variables cannot be stated as causes; only independent variables or parameters play these roles (Kell and Westerhoff, 1986). Many of the correlations given (e.g., Smith et al., 2014) (and they always seem to be given in log.-log. space) have points that are two orders of magnitude apart in one axis. Even if the correlations were valid, they still would not tell us about mechanisms (Kenny et al., 2014); this requires other kinds of experiments. Thus, there are also excellent correlations between the anaesthetic potency of various molecules and their lipophilicity, but equally good correlations exist for the same molecules between lipophilicity and the ability to inhibit luciferase, a soluble enzyme (that is not considered to be involved in mammalian anesthesia as it is not in fact present in mammals) (Franks and Lieb, 1984).

**Figure 3:**
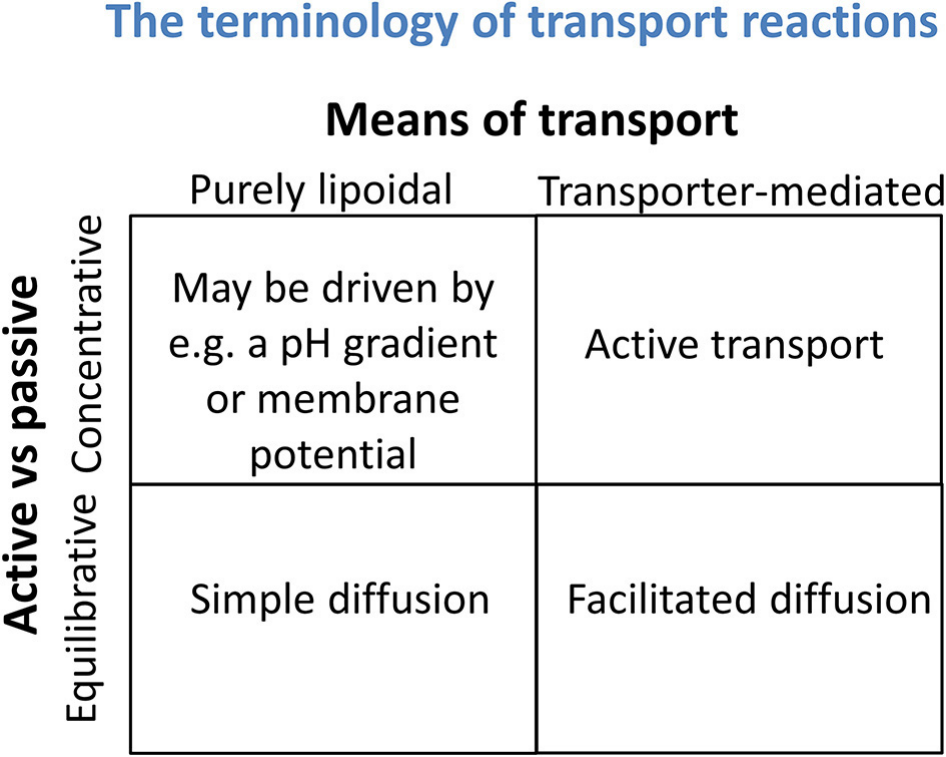
**Transport reactions may be discriminated both by whether they are equilibrative or concentrative in nature (a thermodynamic property) and whether they involve solely any phospholipid bilayer that may be present or instead rely on specific transporters (a mechanistic assessment)**. It is important not to confuse the two.

It is also important to recognize that thermodynamics (and any other state variables) cannot tell you about mechanism either. The “pH-partition theory” (Hogben et al., 1959), of which much is made (e.g., Smith et al., 2014), simply tells one that protonable molecules that can exist at a certain pH in both ionized and non-ionized forms, and that are mainly permeable in the uncharged form, will distribute themselves according to any existing transmembrane pH gradients. This is extremely well-known (and not a recent observation), and such distributions have indeed long been used to *estimate* such pH gradients (Waddell and Butler, 1959), including by us (Kell et al., 1978a,b; Sorgato et al., 1978). Osmotic swelling methods may also be used to estimate the nature (but not the pathway or mechanism) of the most strongly permeating species (Kell et al., 1981). However, again, it is important to recognize that while the absence of a concentration gradient may indicate “the passive diffusion nature of (a transport) process,” the absence of such a gradient does not permit one to conclude whether the transport is through a bilayer by lipoidal diffusion or is carrier-mediated. Thermodynamics can speak to whether a process is passive in nature (i.e., not energy coupled) but not to its molecular mechanism. These two aspects form the orthogonal axes of a “Boston matrix” (Figure 3).

Smith et al. (2014) also repeat claims that a correlation between drug uptake rates of MDCK and Caco-2 cells shows that there must be lipoidal diffusion. This claim is, at best, questionable, when a large fraction of the drugs in the study cited (Irvine et al., 1999) have known transporters (that we have listed previously, Kell et al., 2011).

### Correlation of drug uptake into erythrocytes with log P

**Figure 4:**
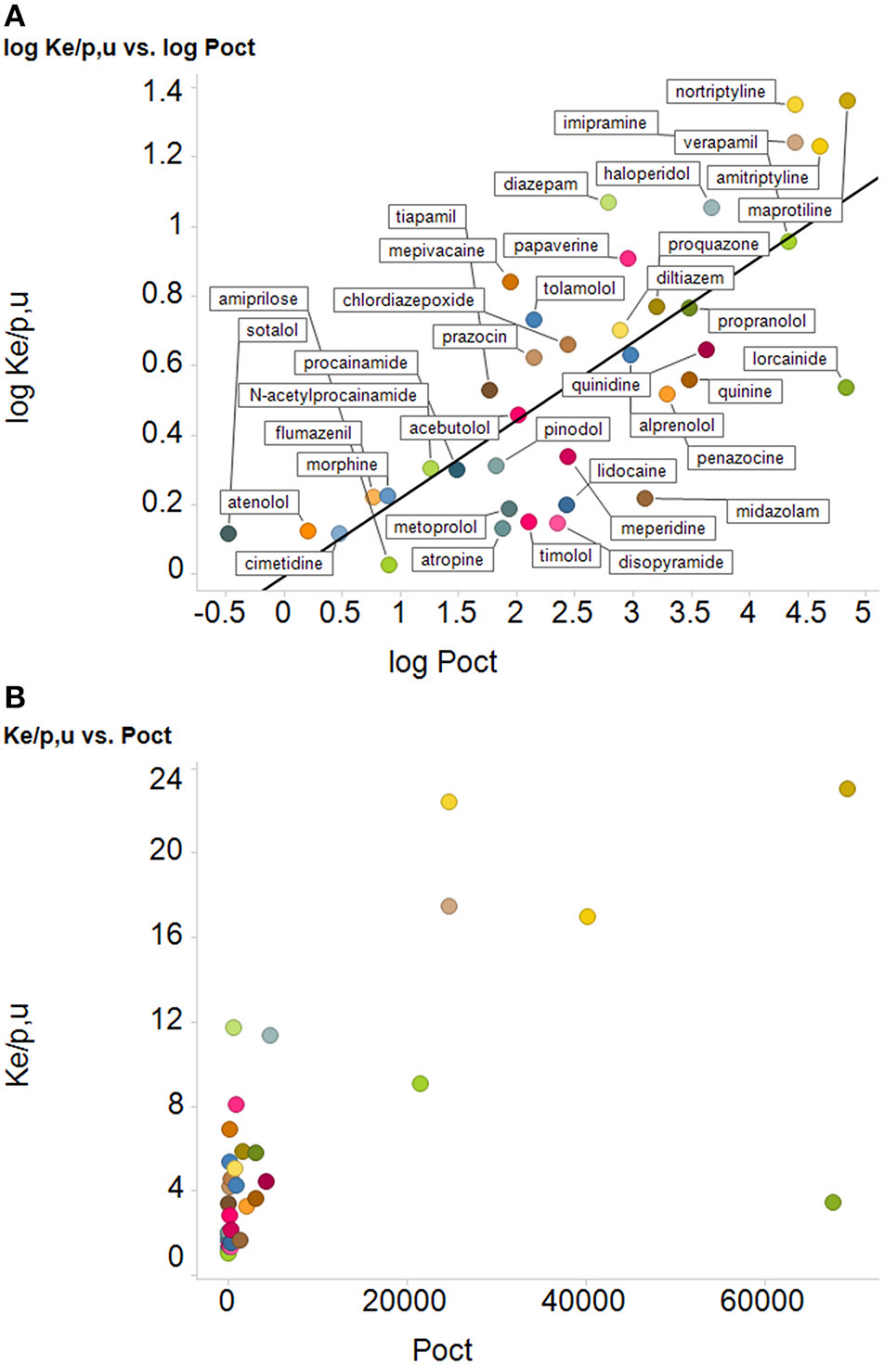
**Relationships between uptake of drugs into erythrocyte and their log P, (A) as redrawn from the plot in Figure 4 of Smith et al. (2014), along with their best-fit straight line (logK_*e/p,u*_ = −0.013 + 0.22 log*P_oct_*), *r*^2^ = 0.59, and (B) the same data plotted with the ordinate encoded linearly, using the same colored symbols as in Figure 4A**. We do not try to fit a straight line through the left-hand 32 drugs and the right-hand 6 drugs.

According to Smith et al. (2014), their “Figure 4 shows that the uptake of drugs into human red blood cells significantly correlates with log P.” We reproduce their Figure 4 as our Figure 4A below. The ordinate data are in fact taken from a review by Hinderling (1997) and an earlier monograph. What is plotted on the ordinate, however, is not the uptake (or partitioning) but (for whatever reason) the *logarithm* of the uptake/partitioning. When we plot erythrocyte uptake against the ability to partition (and not its logarithm) into octanol (Figure 4B), we find that there is, in fact, little correlation. This is unsurprising given that the slope of Figure 4 in Smith et al. (2014) in log-log space is just 0.22 and that some pairs of data points are more than two orders of magnitude away from others with a similar ordinate or abscissa value. We would repeat our advice (Kell et al., 2011) against putting one's faith in log-log plots when their slope is far from unity. Other examples of a lack of correlation of uptake with log P/log D are given below. [Phenomena that do correlate with log P, however, include protein binding (Hughes et al., 2008), drug promiscuity (Azzaoui et al., 2007; Leeson and Springthorpe, 2007; Hann, 2011; Kell et al., 2013) and toxicity (Hughes et al., 2008; Hann, 2011)].

### Inadequate cross-validation of predictive models

According to Smith et al. (2014) “The CNS represents an important vascular/cellular barrier that is accessed in most cases by lipoidal diffusion and is amenable to quantitative structure-permeation relations (Ooms et al., 2002).” Apart from the fact that this is again merely a self-defining assertion (and, see below, because there is little or no paracellular transport, the blood-brain barrier (BBB) arguably represents a system that is in fact *hard* to explain in this way), the paper cited (Ooms et al., 2002) is a very poor example of statistical modeling. A four-latent-variable partial least squares model (effectively a form of correlation) is formed based on (variously) 79–83 objects (compounds) (four inconvenient “outliers” were removed…; Ooms et al., 2002) and 31–72 variables (descriptors), using a version of leave-one-out cross validation. Even with this, the correlation coefficient *r*^2^ *in log-log space* between experimental and predicted values of BBB permeation is only 0.68–0.76 (the slope is not given in the paper), and the *q*^2^ values are just 0.5–0.65. However, this approach to QSAR/QSPR has since been questioned seriously (e.g., Golbraikh and Tropsha, 2002; Cronin and Schultz, 2003; Eriksson et al., 2003; Golbraikh et al., 2003; Tropsha et al., 2003; Broadhurst and Kell, 2006; Tropsha, 2010), and was before (Kell and Sonnleitner, 1995), as the failure to use any kind of *external* validation set (and the paper cited Ooms et al., 2002 did not) makes it extremely prone to over-fitting.

## Intellectual challenges around bilayer lipoidal permeability vs. carrier-mediated drug transport

Smith et al. (2014) raise 15 points that we have made in earlier papers, and offer alternative views. We rehearse these now, since they cover the space of the subject matter quite effectively. The overall aim is (presumably) to find good experiments that will allow us to differentiate between lipoidal diffusion and carrier-mediated transport of different xenobiotics across the membranes of real biological cells, and, where relevant, we shall seek to suggest some based on the 15 points. Later, we suggest others that we think are rather better. In the following, it should be noted that **“statements” and “responses”** come verbatim from Smith et al. (2014), the former are that review's summary of our position and the latter represent its rebuttal of our position; **“counters”** represent this review's answers to the points made in those rebuttals.

**Figure 5:**
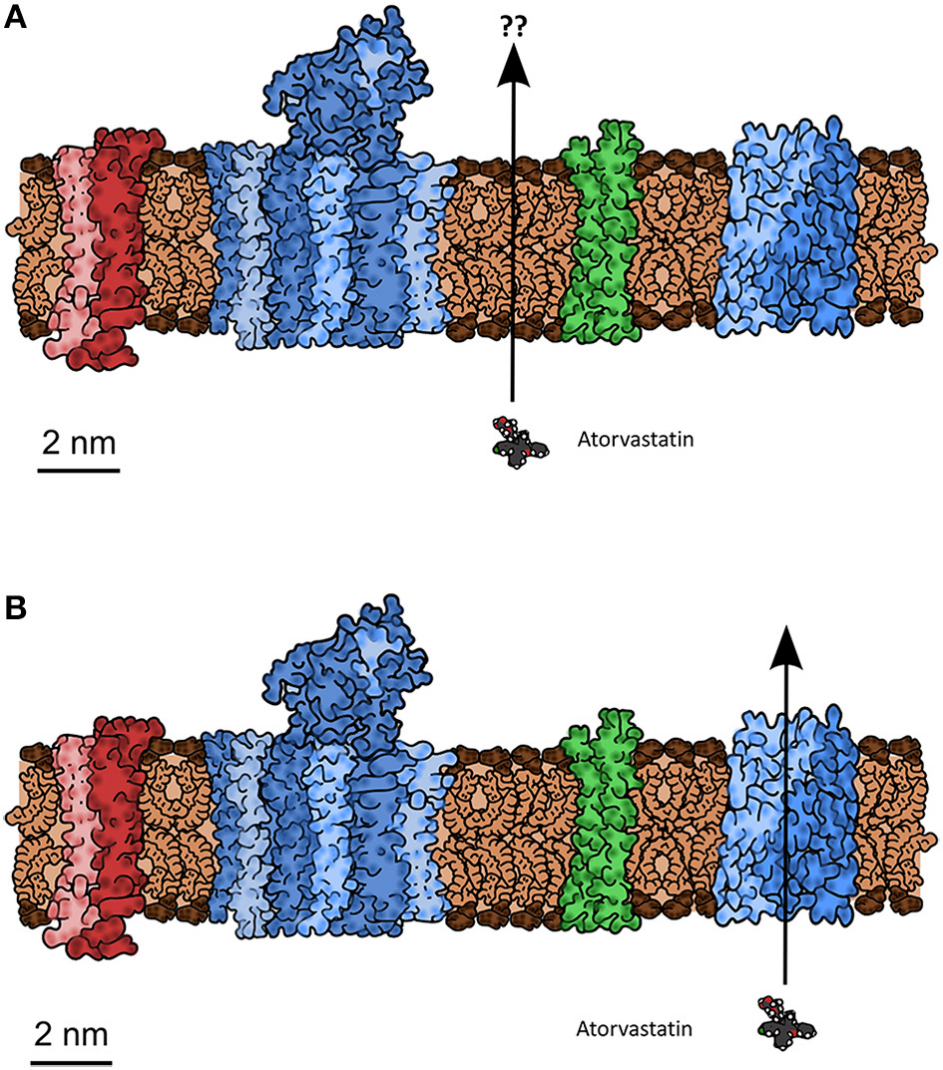
**A typical biomembrane drawn roughly to scale, indicating the typical protein:lipid mass ratio and the possible means by which a small molecule drug (atorvastatin, also drawn to scale) might cross, including (A) bilayer lipoidal diffusion through bilayer areas nominally unaffected by the presence of proteins, or (B) by hitchhiking a lift on transporters (Schlessinger et al., 2013b) normally present for the purposes of intermediary metabolism**. At issue is the question of whether there is any untrammeled bilayer that might let the atorvastatin leak across, and whether biophysical properties such as log D or log P can account for this. Typically atorvastatin is in fact transported by a bile acid transporter known as OATP1B1 (SLCO or SLC21 family) (e.g., Hagenbuch and Stieger, 2013; Higgins et al., 2014).

1. **Statement:** “Lipophilic cations are charged and cannot cross membranes owing to Born charging. **Response**: Drug molecule ions are in equilibrium with neutral non-ionized drug molecules, which have much higher lipophilicity and much higher passive diffusion permeation rate. According to the pH-partition theory, permeation rate varies with solution pH and a compound's pK_a_ such that an increasing ratio of non-ionized/ionized forms correlates with increasing permeation rate.” **Counter**: Smith et al. (2014) seem to miss what we indicated is meant by a lipophilic cation in this context. A typical example given (Dobson and Kell, 2008) is that of the dibenzyldimethylamonium lipophilic cation, that enters yeast via a thiamine transporter (Barts et al., 1980). Lipophilic cations of this type contain quaternary nitrogen atoms with no protons bound directly to the nitrogen; in other words, at any biologically relevant pH, they are always cationic, and they are not “in equilibrium with neutral non-ionized drug molecules.” The same is true for other lipophilic cations of this type, including those we have used—such as butyltriphenylphosphonium (Mccarthy et al., 1981).
2. **Statement:** “The mass ratio of protein:lipid *in vivo* (1/1 to 3/1) affects the transport properties of lipids. Artificial membranes do not model biological membranes, owing to the high protein content *in vivo*. **Response**: These ratios include the cytoplasmic and exoplasmic portions of membrane protein mass, not just the relevant transmembrane fraction. The lipid:protein molar ratio is estimated as 40:1, making lipid an important portion of the membrane exposed to drug molecules. A further refined consideration would take into account the relative cross-sectional area at the membrane surface of the 40 phospholipid molecules to one typical protein. The lipid surface area would still be significantly greater than that of the transporter protein.” **Counter:** The surface area *per se* is not the question. What matters is the extent to which the presence of high amounts of protein in a cell membrane (Dupuy and Engelman, 2008), often binding specific lipids (Laganowsky et al., 2014) (including cholesterol; Song et al., 2014) and certainly altering their organization (Mitra et al., 2004; Engelman, 2005; Mclaughlin and Murray, 2005; Beswick et al., 2011; Coskun and Simons, 2011; Kusumi et al., 2011; Lee, 2011a,b; Domański et al., 2012; KoldsØ and Sansom, 2012; Magalon et al., 2012; Mueller et al., 2012; Smith, 2012; Goose and Sansom, 2013; Javanainen et al., 2013; Van Der Cruijsen et al., 2013) (and *vice versa*; Li et al., 2012; Denning and Beckstein, 2013), alters any ability of drug molecules to cross via the lipoidal bilayer part of the membranes in which these proteins exist. This means that any direct change of lipids will also have the potential likelihood of affecting transporters, so is not of itself a discriminating experiment if transporter activities are not measured. We see an important role for molecular dynamics simulation studies here [see e.g., those of Sansom and colleagues (Stansfeld and Sansom, 2011; Stansfeld et al., 2013), of Tajkhorshid and colleages (Khalili-Araghi et al., 2009; Wang et al., 2010b; Enkavi et al., 2013; Moradi and Tajkhorshid, 2013; Shaikh et al., 2013; Han et al., 2014; Mishra et al., 2014), and of others (e.g., Gedeon et al., 2010; Skovstrup et al., 2012; Denning and Beckstein, 2013; KoldsØ et al., 2013; Schlessinger et al., 2013b)], and note that even CO_2_ can traverse membranes via the central pore in aquaporin (Wang et al., 2010b; Kaldenhoff et al., 2014; Li et al., 2014). It is here worth reminding readers of what membranes actually look like (in cartoon form) (Engelman, 2005), of the size of phospholipid head groups relative to the size of a drug such as atorvastatin (Figures 5A,B), and of the consequent unlikelihood of a drug floating unaided swiftly through a phospholipid bilayer in a real biomembrane.
3. **Statement:** “Correlations of drug uptake with log P and Caco-2 permeation *can* be weak. **Response**: For drugs permeating predominantly by passive lipoidal diffusion, the apparent Caco-2 permeability coefficient, P_app_, can be (and often is) affected by the aqueous boundary layer, filter, paracellular, and lipoidal (transcellular) permeability, as well as the solution pH, as illustrated by the examples in Figure 5 of Smith et al. (2014). log P cannot be directly compared to log P_app_. The Caco-2 intrinsic permeability, log P_0_, is the rational term to compare to log P. P_0_ is easy to deduce from P_app_, but this is seldom done, which often leads to “weak” correlation, as “apple seeds are compared to whole watermelons.” Caco-2 cells from 10 different laboratories were compared in terms of transporter mRNA levels of 72 drug and nutrient transporters, and 17 other targets. It was concluded that “Caco-2 cells from different laboratories produce different results even when using standard protocols for transport studies. The differences may be due to transporter expression as shown for e.g., PepT1 and MDR1 which in turn is determined by the culture conditions. Although the majority of the laboratories used similar culture conditions, absolute expression of genes was variable indicating that even small differences in culture conditions have a significant impact on gene expression, although the overall expression patterns were similar.” Therefore, it is not astonishing that results of Caco-2 cell based permeabilities, when correlated with octanol log P/D values, sometimes show differences in correlations. This is mainly due to the origin and composition of the analyzed data set [ratio actively vs. passively (lipoidal diffusion) transported compounds] and use of partition coefficients (log P) or pH-dependent distribution coefficients (log D). Interestingly, correlations of transport studies performed with different cell lines (e.g., Caco-2/MDCK) commonly used in absorption prediction, with presumably different transporter expression levels, often give excellent correlations, further supporting the coexistence of active and passive transport in biological systems.” **Counter:** So many different things are confused here that it is hard to know what point is actually being made. First, there is the self-defining prophecy (or circular argument) that starts by *asserting* “for drugs permeating predominantly by passive lipoidal diffusion” when this is what we are trying to assess! Then the fact that there is experimental (inter-laboratory) noise in Caco-2 cell measurements is used simultaneously to argue both that it is unsurprising that differences are found, but also that one finds similarities. Finally, there is then a complete jump in logic (“further supporting the coexistence of active and passive transport in biological systems”) that relates concentrative uptake (active) to correlations found in two different cell lines *that each express hundreds of transporters* (Anderle et al., 2004; Landowski et al., 2004; Pshezhetsky et al., 2007; Ahlin et al., 2009; Chen et al., 2010; Volpe, 2011) (and the human genome encodes more; Hediger et al., 2013; Schlessinger et al., 2013b; Viereck et al., 2014). To reiterate: such correlations, if found, can occur regardless of mechanism. When found, and when transport is equilibrative rather than active, they have nothing at all to say about mechanism (whether passive lipoidal or facilitated diffusion, or both, or neither). When they are not found (and there are many examples), one mechanism that can underpin this is carrier-mediated active transport that may occur in some cases but not others depending on the presence of relevant transporters and suitable thermodynamic gradients providing a source of free energy.
4. **Statement:** “Transport across model artificial membranes is stated to occur via pore defects or dissolution in the lipid mixture that are not seen *in vivo*. **Response**: The studies cited are computational simulations (so-called molecular dynamics) of Na^+^ and Cl^−^ ion (non-drug-like) transport under unusual conditions. No convincing experimental evidence for the relevance of pores has been reported. Other experiments indicate the unimportance of pores. Membrane resistance excluding pore diffusion is usually determined by conductivity measurements. Otherwise function of, e.g., ion channels could not be determined.” **Counter:** molecular dynamics (and other) simulations are a highly important part of science (and engineering), and these and other computational analyses will become increasingly so (Hey et al., 2009), not least in systems biology (Kell and Knowles, 2006; Herrgård et al., 2008; Thiele et al., 2013). The non-zero conductivity of e.g., black lipid membranes (Jain, 1972; Tien, 1974; Tien and Ottova-Leitmannova, 2003) can *only* be due to aqueous pores because the Born charging energy is so large that it is inconceivable that Na^+^ and Cl^−^ ions pass through a membrane dielectric with a permittivity of ~2–4 (Parsegian, 1969; Bordi et al., 1999). As stated previously Kell et al. (2013), it is *possible* to make artificial membranes with negligible conductivity [e.g., for biosensors (Aojula et al., 2002), for nanopore sequencing (Bayley, 2006; Stoddart et al., 2009; Rincon-Restrepo et al., 2011), or with Gigaseal patches (Sakmann and Neher, 1984; Neher and Sakmann, 1992)], but this does not mean that experimenters normally do so (and they do not, including ourselves as in a study of liposomal transmembrane proton transport; Kell and Morris, 1980). Moreover, artificial membranes are not biomembranes, which is what we wish to know about.
5. **Statement:** “A dominant role for carrier-mediated transport (and against passive diffusion) is inferred from the hundreds of publications on drug transporters. **Response**: A large number of papers have been published in recent years on transporters. These result from the recent intense research on transporters. However, it is a logical fallacy and a sleight of hand to state that this is evidence of the rate and extent of dominance of carrier-mediated permeation over passive lipoidal diffusion. (An analogy would be to state that newspapers contain a predominance of articles about bad events (e.g., fires, wars, violence, accidents), therefore, bad events dominate good events in the world.) Thus, the large number of citations of publications on transporter research is misleading, because the research they report or review was not undertaken nor concluded by the publication authors as evidence that supports CMOC, as is implied (“There is considerable and increasing evidence that drugs get into cells more or less solely by hitchhiking on carriers normally used for the transport of nutrients and intermediary metabolites).” **Counter:** Most science involves interactions between two important elements, viz. observable data and inferential causation (Kell and Oliver, 2004). One cannot avoid context in discussions of mechanisms. If one observes that the grass is wet that represents an observation or a dataset. However, one cannot infer mechanism simply from an observation (e.g., it was raining vs. someone used a hose to water the garden). The job of the inferential scientist is to take all available data and generalize to the explanation that best accounts for them. This is what we do. Reviews summarizing hundreds of papers can do this in a way that authors of individual papers usually would not. We expect to take most data at face value (albeit some will be wrong), and have a hypothesis that (essentially) says that drugs entering cells always use one or more transporters; (the abundant) data showing the existence of transporters for particular drugs are entirely consistent with that hypothesis (and with PBIN), but have nothing to say about BDII unless bilayer diffusion is actually being *measured directly* (which it is not, in contrast to “good” or “bad” events in the above newspaper analogy, which are) or varied independently (which it is not).
6. **Statement:** “Selected small molecules, urea and glycerol, which cross BLM (bilayer or “black lipid” membranes), permeate to some extent *in vivo* via transporters, except in yeast because glycerol is an osmolyte. **Response**: Urea and glycerol are more hydrophilic than typical drugs that permeate membranes, thus, they are not good models of permeants on which to support theories.” **Counter:** our PBIN hypothesis is entirely general, including for both natural molecules and xenobiotics, and states that there should be transporters even for small molecules (whatever their lipophilicity, and regardless of whether the FDA has approved or not their use as drugs—there is nothing special about drugs *per se*) and that bilayer lipoidal transport is probably negligible. The observable data are consistent with this. *PBIN provides a ready explanation for the lack of membrane permeability of molecules for which the membrane lacks transporters*. This is also true for the BBB, and other tissue and species differences in membrane permeability (see below).
7. **Statement:** “In liposomes the rate of transfer of non-electrolytes depends on MW rather than log P. **Response**: Liposomes correlate well with the permeation behaviors usually observed in artificial and biological membranes. Molecular weight is partly correlated with lipophilicity and hydrogen-bonding capacity, and as molecular weight increases in drugs normally so does hydrogen bonding. Molecular weight is therefore a hybrid term expected to show a relationship to lipoidal membrane permeation.” **Counter:** In fact, MW and log P are rather weakly correlated anyway (e.g., Hughes et al., 2008) and we have no specific preference for either in the absence of an objective function. What we need to be provided with are some predictive hypotheses (see below).
8. **Statement:** “*In vitro* models of diffusion rates across membranes are not based on large sample numbers and validated with compounds not used in the method development. **Response**: This is out of date information.” **Counter:** It is odd merely to refer to this as “out of date information” without providing any evidence, but this could be settled by providing the examples in real biomembranes in which *all* the other relevant transporter-mediated fluxes are removed by deleting the transporter genes (or by any other means).
9. **Statement:** “The flux across *in vitro* PAMPA membranes can be poor even when human absorption is good (e.g., cephalexin, tiacrilast). **Response**: This is out of date information and also is misleading. PAMPA membranes serve to model passive diffusion, whereas cephalexin and a number of other molecules are carrier-mediation transported, as extensively compiled. The present authors claim that passive and active transport processes coexist. PAMPA has been described to only account for passive membrane permeation processes. Therefore, it is not astonishing that actively transported compounds like, e.g., cephalexin cannot be correctly predicted regarding human absorption by methods exclusively focusing on passive transport.” **Counter:** Leaving aside the thermodynamic confusion of “active” and “passive,” we agree that there are many cases for which PAMPA is a poor predictor of the permeability across biological membranes; such cases provide good examples in which passive lipoidal diffusion is not sensibly invoked as a mechanism of xenobiotic transport. (For readers who do not know, PAMPA membranes are various artificial more-or-less hydrophobic barriers that have been used to model transport.) Certainly some molecules can cross protein-free PAMPA membranes very effectively, but this does not tell us about how this relates mechanistically to transport through biological membranes, in which our interests lie. If PAMPA correlates with some calculations based e.g., on log D or other variables, then one could of course use the latter alone to calculate PAMPA behavior if one is interested in it.
10. **Statement:** “Activities of anesthetics were previously thought to be controlled by passive diffusion and correlated to log P, but are now thought to be protein-binding related. **Response**: There is common agreement that drug molecules and anesthetics might interact with proteins, but this is misleading in the context of the discussion, which is around drug transport and not mechanism of action. A recent publication on anesthetics has summarized thus: ‘The molecular mechanism of general anesthesia is still a controversial issue. Direct effect by linking of anesthetics to proteins and indirect action on the lipid membrane properties are the two hypotheses in conflict’.” **Counter:** this is an example of highly selective reporting; the paper cited is not even about biological membranes, and the apodosis of the title of the paper (“looking for a lipid-mediated mechanism of anesthesia”) implies an agenda that seeks to pre-judge the answer. We have discussed the extensive literature of general anesthetics many times (Dobson and Kell, 2008; Dobson et al., 2009a,b; Kell and Dobson, 2009; Kell et al., 2011, 2013; Kell, 2013; Kell and Goodacre, 2014), and the available data show clearly the involvement of a variety of proteins such as GABA_A_ receptors (Mihic et al., 1997; Jurd et al., 2003; Bonin and Orser, 2008), potassium channels (Patel et al., 1999; Thompson and Wafford, 2001; Franks and Honoré, 2004; Gruss et al., 2004; Heurteaux et al., 2004; Grasshoff et al., 2006; Andres-Enguix et al., 2007; Bertaccini and Trudell, 2012), glycine receptors (Mihic et al., 1997; Lobo and Harris, 2005; Dickinson et al., 2007; Bertaccini et al., 2010), and NMDA receptors (Sanders et al., 2003; Dickinson et al., 2007; Dickinson and Franks, 2010). The sites of interaction of general anesthetics with a number of their target membrane proteins are now known with molecular resolution, including their variation in mutant forms of the same proteins (that correlate with changes in anesthetics potency—see e.g., Nury et al., 2011; Stansfeld and Sansom, 2011). Such evidence makes clear precisely what the protein targets of general anesthetics are. We invoke the story of the changes over time in our understanding of the mechanisms of narcosis (general anesthesia) because the whole discussion is *precisely* about the mechanism of action (i.e., transport) of drugs crossing membranes, and because this purportedly (according to Smith et al. (2014) and others) occurs passively through bilayers according to their lipophilicity, just as was once believed for general anesthetics. The analogy is both appropriate and clear-cut, and the change in understanding over time is likely to be of a similar nature.
11. **Statement:** “Many molecules (e.g., ethanol) have relatively specific receptors, so they may have similar protein binding (unidentified) that effects membrane permeation. **Response**: This is an assumption and generalization awaiting to be proven by experimental data, but which currently does not rule out transport by passive (lipoidal diffusion) mechanism.” **Counter:** As explained, the Popperian view does not allow one to rule out anything for which a specific mechanistic hypothesis has not been given, nor to “prove” it, and the bilayer lipoidal diffusion hypothesis for intact biomembranes is not set down in a properly testable way. By contrast, PBIN states that binding proteins and transporters will be found for all kinds of molecules. Another recently discovered binding protein for ethanol [additional to GABA_A_ receptor subtypes (Wallner et al., 2003, 2006; Nutt et al., 2007; Olsen et al., 2007; Santhakumar et al., 2007; Bonin and Orser, 2008; Lobo and Harris, 2008; Mody, 2008; Meera et al., 2010; Johnson et al., 2012) and many others where the binding site is known to atomic resolution (Howard et al., 2011b)] is GLIC, a prokaryotic member of the pentameric ligand-gated ion channel (pLGIC) family (Stansfeld and Sansom, 2011; Howard et al., 2011a, 2014). Other recent papers on ethanol-binding proteins include ones on Munc13-1 (Das et al., 2013) and alcR (Sakvarelidze et al., 2007) (which was discovered in 1985; Doy et al., 1985), so ethanol receptors are not “unidentified.” The final identification of e.g., yeast ethanol *transporters* is not yet certain, but assessing the contributions of such membrane proteins to solvent tolerance is one experimental approach to detecting them (Kieboom et al., 1998a). While other mechanisms are also possible (Dikicioglu et al., 2014), the ABC transporter (Sá-Correia et al., 2009) Pdr18 (Teixeira et al., 2012) and the glyceroaquaporin Fps1 (Teixeira et al., 2009) have properties consistent with such a role as ethanol transporters in yeast, a fact of considerable biotechnological relevance (Dunlop et al., 2011). In the context of biofuels production (and ethanol is a biofuel), and based on similar strategies of toxicity resistance to the one that we exploited earlier (Lanthaler et al., 2011), we now also know them for a variety of other rather lipophilic substances such as alkanes (Tsukagoshi and Aono, 2000; Fernandes et al., 2003; Ankarloo et al., 2010; Chen et al., 2013; Doshi et al., 2013; Foo and Leong, 2013; Ling et al., 2013; Nishida et al., 2013), arenes (Kieboom et al., 1998b), terpenoids (Jasiński et al., 2001; Yazaki, 2006; Foo and Leong, 2013), long-chain fatty acids (Wu et al., 2006a,b; Khnykin et al., 2011; Lin and Khnykin, 2014; Villalba and Alvarez, 2014), short-chain fatty acids (Gimenez et al., 2003; Islam et al., 2008; Moschen et al., 2012; Sá-Pessoa et al., 2013), etc. These are all substances for which bilayer lipoidal diffusion was “once widely assumed” (and presumably still is in some quarters). Ethanolamine transporters are well-established in certain salmonellae (Stojiljkovic et al., 1995; Penrod et al., 2004).
12. **Statement:** “Carrier-mediated drug uptake is observed where it has been studied. (Presumably this circumstantially indicates that transporters will be found for all drugs.) **Response**: Carrier-mediated drug uptake may be observed, but it may not account for 100% of the transport. In Michaelis-Menten analysis, the non-saturable term usually is related to the passive diffusion contribution.” **Counter:** reiterating the fact that something (the transport via carriers you do not know about) does not exist because you have not found it is illogical. See the section about absence of evidence not being evidence of absence. By contrast, PBIN is testable because we make specific predictions about drug transporters and the effects of removing them (by deleting their cognate genes) or increasing their activities (and, yes, we consider it likely that transporters will be found for all drugs). Saturability is not a useful criterion. One would hardly deny the existence of aquaporins because of the fact that they may not be observably saturable in the experimentally testable range.
13. **Statement:** “Drugs can concentrate in specific tissues beyond the stoichiometry of internal binding sites. This phenomenon absolutely requires an active uptake process. **Response**: This can be due to pH gradients between intracellular and extracellular compartments as described for, e.g., basic amines and safety relevant lysosome accumulation (phospholipidosis).” **Counter:** agreed, see above; this is well-known (and a pH gradient can provide a thermodynamic drive that could cause transport to be active, i.e., concentrative, whatever the mechanism of transmembrane transfer). The same general idea is true (in principle) for charged molecules or those whose transport is ion-coupled accumulating (or not; Kell, 1992) in compartments where there is a membrane potential difference relative to elsewhere. These are thermodynamic statements, not mechanistic ones.
14. **Statement:** “Biophysical forces in drug-lipid membrane interactions (e.g., lipophilicity, hydrogen bonding) are no different from drug-protein interaction. Thus, physicochemical properties and the rule of 5 need not be evidence of passive diffusion. **Response**: Of course biophysical forces apply to both transporters and bilayers. However, the physical property differences between the rate limiting barriers for a particular drug in carriers and bilayers can dictate the predominant route of transport. A second argument is that ligand-protein recognition is dominated by highly selective stereoelectronic features far more than by global (molecular) physicochemical properties.” **Counter:** One potential approach to discriminating bilayer lipoidal diffusion from transport via proteins with similar biophysical characteristics is to compare their biophysical characteristics and make and test some specific predictions. The effects are likely to be subtle, but may be measurable. We invite the proponents of BDII to do this, if they can work out a means for actually measuring (as opposed to assuming) bilayer lipoidal diffusion in real biological membranes. However, arguments based on the substrate specificity of enzymes, when you do not even know them, are utterly pointless. We have tried to explain several times (e.g., Kell et al., 2011, 2013) the extremely well-known fact that some enzymes are highly promiscuous (and see e.g., O'brien and Herschlag, 1999; Hopkins et al., 2006; Ma and Lu, 2008; Nobeli et al., 2009; Carbonell and Faulon, 2010; Khersonsky and Tawfik, 2010; Gatti-Lafranconi and Hollfelder, 2013). If an enzyme uses a great many substrates it is not very likely that it will be very discriminative of e.g., stereoisomers of the *same* molecule [although some transporters are stereoselective (Zhou et al., 2014), e.g., those for propranolol (Wang et al., 2010a; Zheng et al., 2013)]. By contrast, the activity of many promiscuous enzymes (e.g., the cytochromes P450, e.g., O'reilly et al., 2011, 2013; Munro et al., 2013) can be related (up to a cut-off) to the lipophilicity of the substrate. This is very simply explained in terms of the possession of a hydrophobic substrate pocket.
15. **Statement:** “The notion of passive lipoidal permeation is traced back to artificial membrane systems, which are not successful predictors of membrane permeation. **Response**: On the contrary, artificial membrane models have been successful predictors of passive lipoidal permeation.” **Counter:** although not especially relevant, the notion of passive lipoidal permeation is in fact traced back to long before the invention of model membrane systems, or even our knowledge (Gorter and Grendel, 1925) of the bilayer thickness of biological membranes; Smith and colleagues do in fact cite the work of Overton in 1899 that initiated it (Overton, 1899). We have dealt with correlations enough.

## Testable hypotheses: experiments that may be useful for discriminating PBIN and BDII

The importance of transporters to the uptake of existing clinical drugs is a backward-looking enterprise, but we have previously given a variety of examples of drugs, such as the (Lipinski-compliant; Lipinski et al., 1997; Lipinski, 2004) nucleoside anti-pancreatic cancer drug gemcitabine, that clearly are efficacious only when transported by relevant transporters (e.g., Mackey et al., 1998, 1999; Rauchwerger et al., 2000; Huang and Sadée, 2003; Mangravite et al., 2003; Huang et al., 2004; Spratlin et al., 2004; Giovannetti et al., 2006; King et al., 2006; Marcé et al., 2006; Mori et al., 2007; Nakano et al., 2007; Oguri et al., 2007; Molina-Arcas et al., 2008; Andersson et al., 2009; Farrell et al., 2009; Maréchal et al., 2009, 2012; Hagmann et al., 2010; Molina-Arcas and Pastor-Anglada, 2010; Bhutia et al., 2011; Komori et al., 2011; Parkinson et al., 2011; Santini et al., 2011; Wang et al., 2011; Borbath et al., 2012; Damaraju et al., 2012, 2014; Gesto et al., 2012; Murata et al., 2012; Ansari et al., 2013; Jordheim and Dumontet, 2013; Nakagawa et al., 2013; Skrypek et al., 2013; Xiao et al., 2013; De Sousa Cavalcante and Monteiro, 2014; Lee et al., 2014; Nordh et al., 2014; Tong et al., 2014). We do not know of any evidence that gemcitabine (or any other nucleoside) exhibits *any* significant bilayer lipoidal diffusion across intact cellular membranes, although the question of whether a molecule is “thought to permeate mainly by passive lipoidal diffusion” does of course depend on who is doing the thinking.

We stress that our analyses are based on all kinds of molecules, whether the FDA has approved them as drugs or not. Consequently, we shall use as examples clinical drug candidates and other xenobiotics, as well as marketed drugs.

## Testing the PBIN view

Smith et al. (2014) made some suggestions as to how the PBIN view may be tested. Rather than quoting them verbatim, we summarize the relevant topics.

### Identifying relevant transporters

This is very important, and websites such as Transportal (Morrissey et al., 2012) http://bts.ucsf.edu/fdatransportal/, DrugBank (Law et al., 2014) http://www.drugbank.ca/ and others reviewed in Viereck et al. (2014) contain literally hundreds of examples in which known drugs use known transporters, complete with quantitative data, sometimes for genetic variants that change activity or expression. While the BDII view merely *assumes* lipoidal transport and varies nothing systematically to try to assess it, PBIN makes specific predictions via causing variation in the activities of specified transporters, and the starting point is that one should find out which they are.

To this end, we would cite the work of Brummelkamp, Superti-Furga and colleagues, who have developed a near-haploid mammalian cell line (Carette et al., 2009, 2011; Bürckstümmer et al., 2013) along with a suitable retrovirus (actually a gene trap (GT) retrovirus) that can insert into more-or-less any gene, thereby inactivating it. In a manner similar to that which we used in yeast (Lanthaler et al., 2011), they have been studying the efficacy of an anticancer drug, sepantronium bromide (also known as YM155, see e.g., Giaccone et al., 2009; Nakahara et al., 2011; Aoyama et al., 2013; Murakami et al., 2013), in various cell lines. They find (Winter et al., 2014) (and cf. Minematsu et al., 2009, 2010) that the uptake of this drug, and thus its ability to kill mammalian cells, essentially depends *entirely* (and quantitatively) on the expression of a single, specific transporter, viz SLC35F2 (see Ishida and Kawakita, 2004; Song, 2013). They find precisely *no* evidence for *any* lipoidal diffusion (nor of efflux transporters). This general method can only really “work” (i.e., serve to illuminate the transporters effecting significant fluxes of cytotoxic or other drugs) if the “non-specific” background rate (e.g., via bilayer lipoidal transport) is negligible, and will clearly be of very great utility in discovering precisely which drugs use which transporters (Bassik and Kampmann, 2011; Reiling et al., 2011; Bürckstümmer et al., 2013).

In a similar vein, cloned or recombinant transporters are a very useful strategy (and one widely used, e.g., Mackey et al., 1999; Srimaroeng et al., 2008; Giacomini et al., 2010; Brouwer et al., 2013; Winter et al., 2014) but, as mentioned above, saturability, the availability of inhibitors or the extent of promiscuity or otherwise are not at all discriminatory (Kell et al., 2011). *Much better criteria* relate to varying the activities or expression levels genetically (e.g., by cloning transporters—independent variable) and then seeing the consequent effects of their expression levels (here an independent variable) on drug transport (dependent variable). QConCats (e.g., Pratt et al., 2006; Rivers et al., 2007; Brownridge et al., 2011; Carroll et al., 2011; Achour et al., 2014; Chen and Turko, 2014) provide an important (Harwood et al., 2013) and absolute means of measuring expression levels of target proteins, including transporters (Russell et al., 2013), while other absolute approaches to transporter quantification are also emerging (Ohtsuki et al., 2011, 2013; Uchida et al., 2011b, 2013; Obuchi et al., 2013; Qiu et al., 2014).

In a similar vein, the pH-dependence of uptake and pH-partition theories are both very hard to interpret and essentially irrelevant to the question of mechanism; the latter depends solely upon which species happens to be most permeable (and they are not always those expected; Mazák and Noszál, 2014).

### Varying transporter expression in established cell lines

Worthwhile experiments on this will be doable using genetic knockouts, gene traps, or siRNA, etc. Fortunately we know many of the relevant transporters from genome sequencing, and the expression of *hundreds* of proteins in the membranes of MDCK (Chen et al., 2010) and Caco-2 (Anderle et al., 2004; Landowski et al., 2004; Pshezhetsky et al., 2007; Ahlin et al., 2009) cells are known from transcriptome and proteome studies. The example of SLC35F2 is very pertinent—the recognition of its activity in transporting sepantronium is new (Winter et al., 2014) but the transporter (albeit not its natural substrate) was known (Ishida and Kawakita, 2004; Song, 2013). Thus, we can now predict that the transport of sepantronium into Caco-2 or MDCK will depend upon the activity of a transporter that will likely be the same as or homologous to SLC35F2.

### Varying lipoidal diffusion as an independent variable

For those who believe that BDII, the hypothetico-deductive approach requires that one varies it as an independent variable and/or measures it as a dependent variable. Since we believe that passive lipoidal bilayer diffusion in real biomembranes is initially negligible, and give many examples, it is not obvious how we could slow it down! However, we would stress that broad changes such as e.g., temperature will affect both lipids and transporters and are not suitably discriminatory (especially if transporter fluxes are not measured). Neither is changing lipid composition alone discriminatory, since (see above) changes in lipids can have profound effects on the activities of membrane proteins, including transporters. So, to be discriminatory it is necessary to measure any such effects on known and relevant transporters as a control. We have also explained many times that only tests in real membranes can tell us what is happening in real membranes, and that there is no “*observed* passive lipoidal permeation of biological membranes,” only an *inferencing* of it. It is also important to make well-defined comparisons with a given species and cell type or line. Species differences (see below) can be enormous, let alone differences between real biological membranes and model membranes lacking proteins. Adding small amounts of lipids that can be made to crosslink to each other but not to proteins (nor to bind to them) may or may not be informative.

## We propose some candidate discriminating experiments that adherents of the BDII theory and others might care to perform or assess

According to our reading, the BDII view allows all kinds of xenobiotics to cross biomembranes, by diffusing through whatever phospholipid bilayer portions that they may contain, thereby equilibrating their internal and external concentrations according to whatever thermodynamic forces may be operating, regardless of cell, tissue, individual, or species. A considerable number of corollaries follow from this BDII view. We think that the existing data do not follow those corollaries, at least without adding *ad hoc* and extra hypotheses to make special cases. However, it will be important to be clear as to precisely what the adherents of the BDII theory claim to be true in a testable manner, so we can evaluate whether the data are or are not consistent with these predictions. We make some suggestions as to where discriminatory experiments are likely to serve.

### What is the predicted relationship for the BDII theory between log D and fluxes across real biomembranes?

According to Smith et al. (2014), “Passive lipoidal permeability is correlated positively with lipophilicity (e.g., as expressed by the log of the octanol-water partition, log P, or the apparent value at a given pH, often 7.4, log D).” Such a statement requires that we have a precise prediction as to the *form* of this relationship over a stated range of values of log P and log D (and state which variant of log P is used if it is calculated, and with which software so that this may be reproduced).

**Figure 6:**
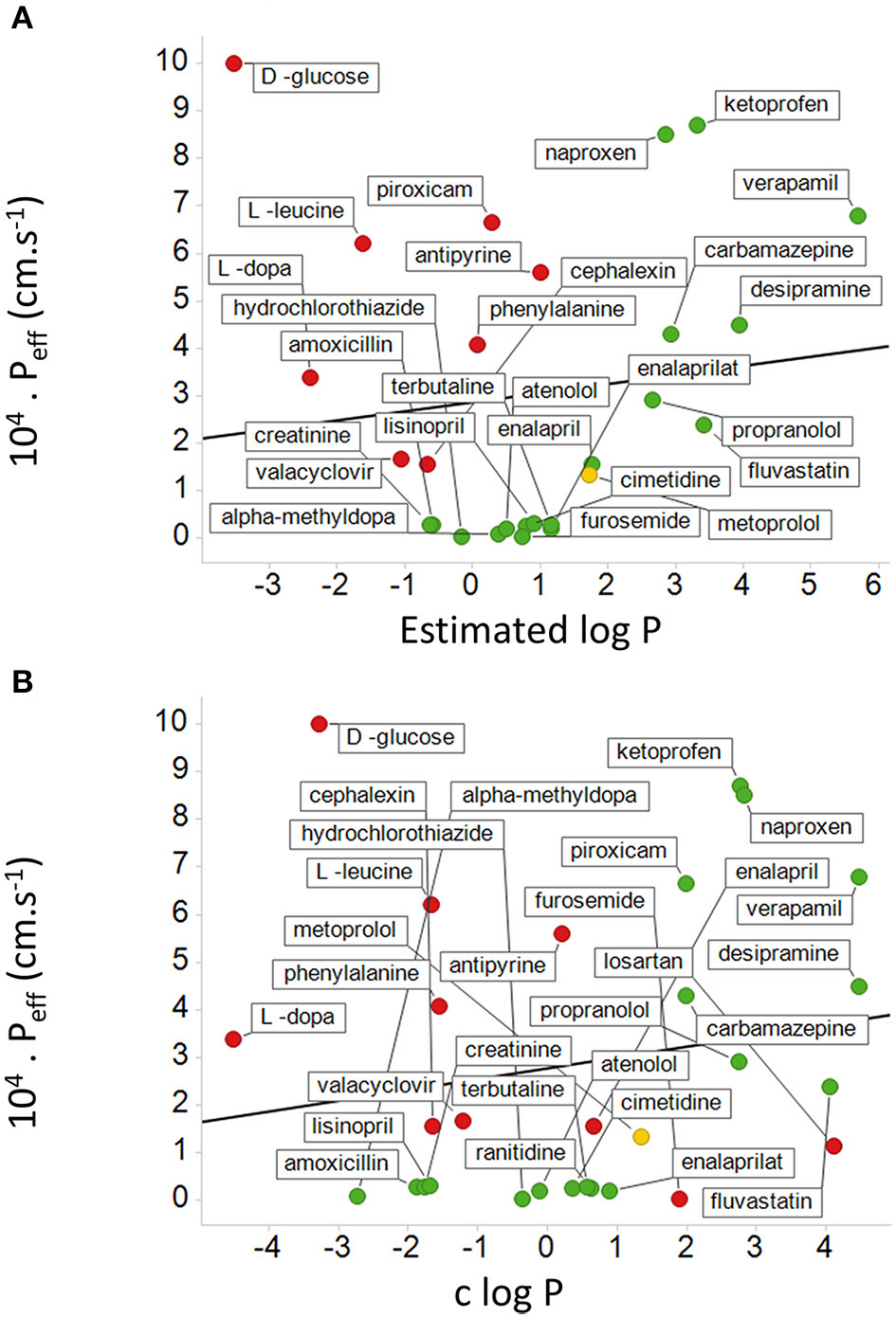
**Relationship between measured human jejunal permeability and log P**. The abscissa is either based on **(A)** an estimated log P or **(B)** a calculated log P (c log P). Data are re-plotted from Table 4 and Figures 4, 5 of Kasim et al. (2004). In **(A)** data are not available for losartan and ranitidine, and there are 8 false negatives shown in red. Metoprolol is a “reference compound” (Kasim et al., 2004; Incecayir et al., 2013; Zur et al., 2014) and is shown in yellow. In **(B)** there are also two false positives. The lines shown are the lines of best fit; in **(A)** the correlation coefficient is 0.12 while in **(B)** the correlation coefficient is 0.18.

**Figure 7:**
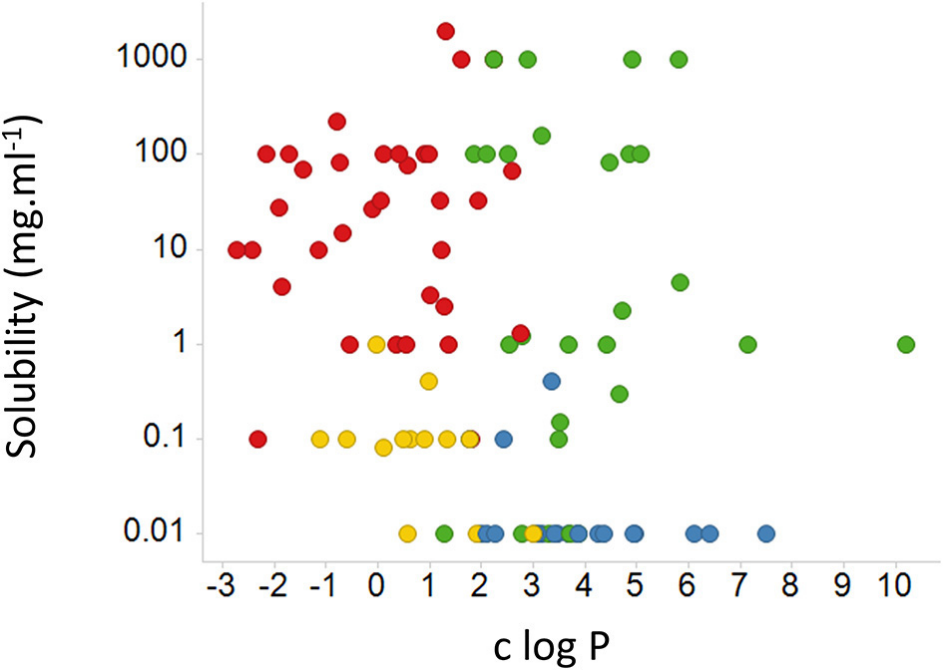
**Relative lack of relationship between the aqueous solubility of a drug and c log P for various drugs, marked by their BCS classes**. We used pdfx (Constantin et al., 2013; http://pdfx.cs.man.ac.uk) to extract the data from Table 2 of Kasim et al. (2004). BCS class is encoded in the color of the symbols: 1, green; 2, blue; 3, red; 4, yellow.

While it is not obvious which *actual measurements* (as opposed to assumptions) of passive lipoidal permeability in biological membranes are being claimed (and we know of none), the above statement would also predict that if lipoidal bilayer permeability of drugs were a dominant means of drug uptake there should thus be a good correlation between *cellular uptake* and log P. This is a very important and testable prediction. Our very first review (Dobson and Kell, 2008) displayed a typical example taken from a paper by Corti et al. (2006), showing that there is not, and we would like to stress that this paper was not specifically selected—it just happened to be the first paper we looked at for this question. We here discuss another, rather famous, dataset. The Biopharmaceutics Classification System (BCS), based on the work of Amidon and colleagues (e.g., Amidon et al., 1995; Dahan et al., 2009; Chen et al., 2011), was developed to indicate a “bioequivalence,” and divides drugs into four classes based on their solubility and presumed (human jejunal) permeability, with “class 1” drugs that display high solubility and permeability deemed favorable and a waiver given http://www.fda.gov/AboutFDA/CentersOffices/OfficeofMedicalProductsandTobacco/CDER/ucm128219.htm (and see Lennernäs et al., 2014). The experimental jejunal permeability is not always available, and so it is estimated based on a “correlation” between the permeability of a drug's neutral form and c log P determined for a small number of drugs. The data for 27 or 29 such drugs vs. “estimated log P” and c log P are re-plotted from Table 4 and Figures 5, 6 of Kasim et al. (2004) (who very helpfully provided data in both tabular and graphical forms) in our Figure 6. We note there the extremely modest extent of the correlation between the experimental permeability and either the “estimated log P” or c log P. We also note that six of the eight “false negative” drugs (D-glucose, L-leucine, L-Dopa, L-phenylalanine, cephalexin, and valacyclovir) that were predicted to have low permeability but in fact had high permeability were recognized by the original authors as having transporters (Kasim et al., 2004). It is not clear whether it was assumed that the other drugs in Table 4 of Kasim et al. (2004) crossed passively by lipoidal bilayer diffusion (as opposed to facilitated diffusion through a transporter, that is also passive, Figure 3). At all events, whether it was so assumed or not, we can find evidence for interactions with transporters for each of the other drugs except for antipyrine, carbamazepine, and terbutaline. These are: α-methyldopa (Uchino et al., 2002), amoxicillin (Li et al., 2006; Sala-Rabanal et al., 2006; Fujiwara et al., 2011, 2012), atenolol (Kato et al., 2009), cimetidine (Collett et al., 1999; Burckhardt et al., 2003; Motohashi et al., 2004; Pavek et al., 2005; Matsushima et al., 2009; Tsuda et al., 2009), creatinine (Schömig et al., 2006; Chen et al., 2009; Zhou et al., 2009; Hosoya and Tachikawa, 2011; Tachikawa and Hosoya, 2011; Torres et al., 2011), desipramine (Wu et al., 2000; Haenisch et al., 2012), enalapril (Pang et al., 1998), enalaprilat (Ishizuka et al., 1998), fluvastatin (Varma et al., 2011; Sharma et al., 2012), furosemide (Uwai et al., 2000a; Eraly et al., 2006; Vallon et al., 2008), hydrochlorothiazide (Race et al., 1999; Uwai et al., 2000a; Hasannejad et al., 2004; Han et al., 2011), ketoprofen (Khamdang et al., 2002; Morita et al., 2005), Lisinopril (Knütter et al., 2008), losartan (Edwards et al., 1999; Race et al., 1999; Knütter et al., 2009; Sato et al., 2010), metoprolol (Dudley et al., 2000), naproxen (Apiwattanakul et al., 1999; Mulato et al., 2000; Khamdang et al., 2002; El-Sheikh et al., 2007), piroxicam (Jung et al., 2001; Khamdang et al., 2002), propranolol (Dudley et al., 2000; Wang et al., 2010a; Kubo et al., 2013b; Zheng et al., 2013), ranitidine (Collett et al., 1999; Müller et al., 2005; Ming et al., 2009), and verapamil (Döppenschmitt et al., 1999; Kubo et al., 2013a). We have also plotted (Figure 7) data from the Oral Drugs in the Core WHO Essential Medicines List (Table 2 of Kasim et al., 2004). These show the essential lack of a major relationship between solubility and c log P (and neither is well-correlated with bioavailability; Sutherland et al., 2012). A more recent predictive modeling study (Ghosh et al., 2014), in which the word “transporter” does not appear once, developed a theoretical framework for “passive permeability” and applied it to nine substances; these are, with some references indicating that they each have known transporter interactions, as follows: testosterone (Hamada et al., 2008; Sharifi et al., 2008), warfarin (Marchetti et al., 2007), dexamethasone (Polli et al., 2001; Schwab et al., 2003; Uchida et al., 2011a), raffinose (Tyx et al., 2011), metoprolol (Dudley et al., 2000), propranolol (Wang et al., 2010a; Zheng et al., 2013), verapamil (Döppenschmitt et al., 1999; Kubo et al., 2013a), ibuprofen (Uwai et al., 2000b) and (the lipophilic cation) crystal violet (Burse et al., 2004a,b).

**Figure 8:**
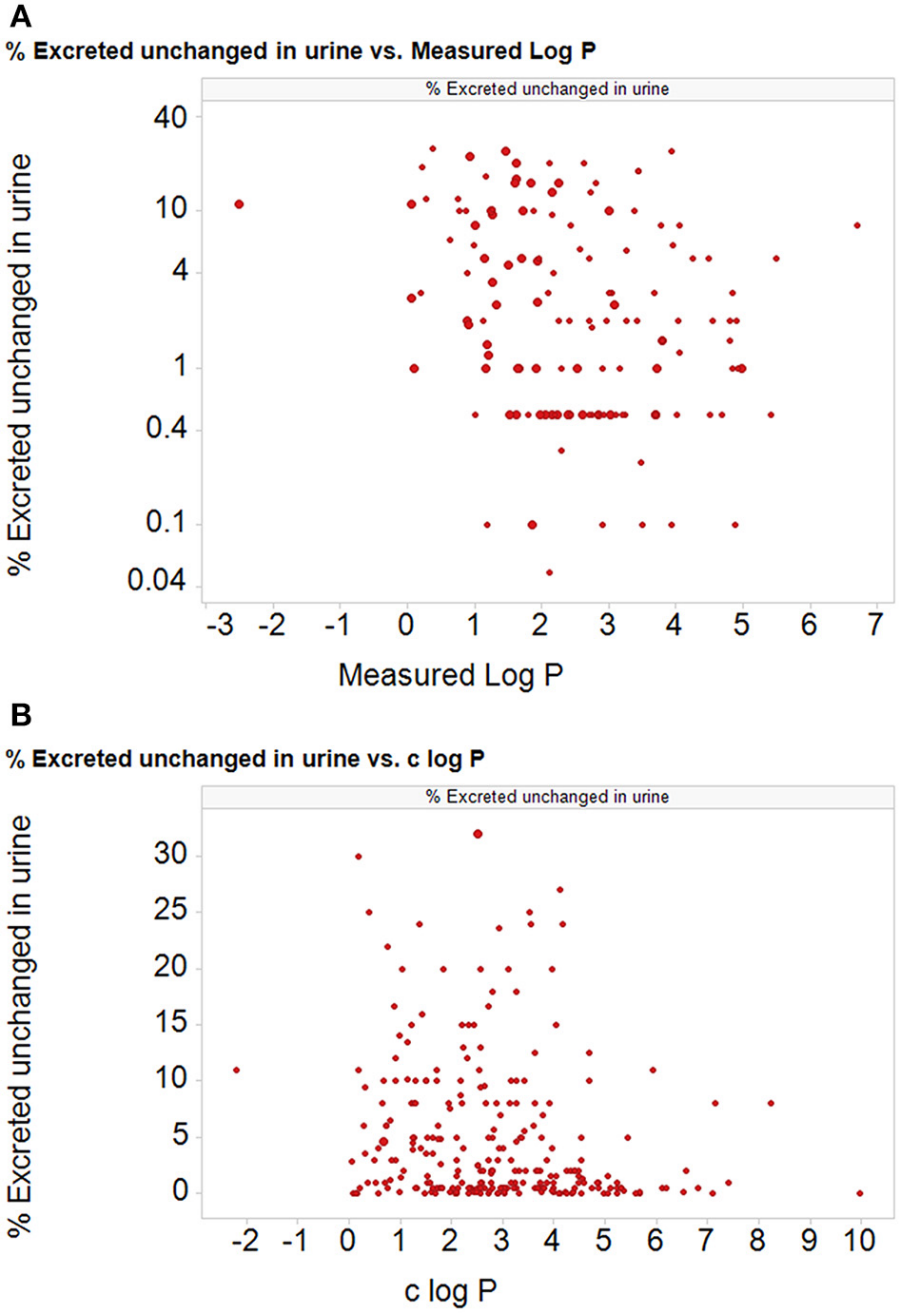
**The percentage of particular drug doses excreted in the urine for 350 of 351 “class 1” BDDCS drugs (Benet et al., 2011; one drug was excluded as it was too water soluble to measure) as a function of (A) the measured and (B) the calculated log P (c log P) (calculated using the RDKit software Landrum et al., 2011 and KNIME, Berthold et al., 2007; Mazanetz et al., 2012)**. Ordinate data are taken from Table 1 of Benet et al. (2011).

According to the Biopharmaceutics Drug Disposition Classification System (BDDCS) (Benet et al., 2008, 2011; Benet, 2010), which bears at least some similarities to the BCS, the disposition of drugs represented in its class 1 (high permeability and high metabolism) category is considered to be completely unaffected by the presence of transporters in the gut and liver. At least two interpretations of this are possible (Estudante et al., 2013): (i) there are no transporters interacting with these drugs and all the transport is by lipoidal diffusion, or (ii) there are *so many* high-flux transporters that they simply do not provide a barrier to uptake. A surrogate for cellular uptake and metabolism in the BDDCS system is the extent to which drugs are excreted unchanged in the urine (low extent unchanged implying high metabolism, hence cellular uptake), and we have redrawn (Figure 8) plots of this against both measured and calculated log P values for 350 of the 351 BDDCS class 1 drugs tabulated (rather than being visualized) in Benet et al. (2011). It is obvious that the amount of drug excreted unchanged in the urine (and thus presumably its cellular permeability) can take almost any value whatever the value of log P, over an extremely wide range of values of log P. We have not chosen to fit a statistical line to either of these figures. Thus, we also suggest that it is useful if data that are supposed to support claims are made available in both tabular and graphical form, the latter with linear coordinates on both axes.

### Which metabolites are supposed not to have transporters?

Supporters of BDII regularly make claims about molecules that are supposed to be transported by bilayer diffusion, without telling readers which molecules they are. However, many of those that are stated to use bilayer lipoidal diffusion do in fact have known transporters, which thus makes any discrimination impossible. Presumably supporters of BDII have some ideas about biological systems and/or drugs for which they consider that there is *no* transporter acting on the molecule in question. It would be helpful if those who believe that BDII were to provide a reasonably extensive list of molecules (including marketed drugs) that (on whatever stated criteria) are supposed to be transported solely via bilayer diffusion so that those who expect to find suitable transporters can seek them. Note the evidence we gave above for transporters for quite lipophilic molecules, including alkanes.

We also note that there is much available online data and evidence of drugs that have known transporters, for instance at Transportal (Morrissey et al., 2012) and DrugBank (Law et al., 2014).

### Criteria that might reasonably be required to indicate that a drug is a substrate for a particular transporter?

Smith et al. (2014) bring up for discussion whether the identification of some drug transporters was conducted with due “rigor and precision,” but the nature of their objection is unclear. In microbiology, it is common to use Koch's postulates (see Kell et al., 1998) to argue that microbe X is the “cause” of disease Y. In a similar vein in molecular genetics, one usually takes it that to claim that gene (product) X is causative (at least in part) of phenotype Y, the removal or change in activity of gene (product) X as an independent variable should have predictable effects on phenotype Y. If we claim that transporter X transports drug Y (and it may also be annotated as being a transporter of natural metabolite Z) the conventional “rigor and precision” is to vary (including to zero) the activity of gene product X, whether by genetic means or otherwise, and observe the effects on (the transport of) Y. If it is considered from known arguments that metabolite Z is also a substrate (or inhibitor) of transporter X then the prediction is that adding Z will decrease the (contribution of transporter X to the) uptake of drug Y, according to standard enzyme kinetic mechanisms (Keleti, 1986; Cornish-Bowden, 1995; Fersht, 1999). This is *precisely* what was done in papers such as (Lanthaler et al., 2011; Winter et al., 2014). It would be valuable if supporters of BDII would provide any arguments that state that these are not seen as proper criteria for claiming, or at least contributing substantially to a claim, that a particular drug is transported by a particular, genetically identified, transporter, as well as any criteria that can be applied with the same logic or rigor to the assessment of phospholipid bilayer uptake.

Overall, these kinds of rigorous, genetically modulated changes leading to predictable outcomes contrast entirely with statements that observable phenomena are caused by bilayer diffusion when there has been no attempt to *modulate that as an independent variable* nor to *measure it directly*. As stated above, however, we note that changing lipids *per se*, without knowing about their contingent effects on transporter proteins at the same time (see references on protein-lipid interactions, above), is not a suitably discriminatory experiment.

### How does BDII account for the “blood-brain barrier” (and other such “barriers” within an organism), without invoking efflux transport reactions that have not been measured?

As is well-known, many (if not most) drugs fail to cross the BBB (Pardridge, 2012), despite the fact that brain lipids are not thought to differ substantively from lipids in other tissues. Certainly paracellular routes that exist in other tissues are not apparently available at the BBB, which helps to sharpen the arguments. Leaving aside efflux transporters (Bagal and Bungay, 2014), PBIN has no trouble explaining this in terms of a relative lack of suitable transporters at the BBB—indeed a lack of permeability in their absence is *expected*. (a) for drugs for which efflux transporters at the BBB are not known, it would be useful to know how proponents of the BDII theory explain the virtually complete lack of uptake of those drugs, including lipophilic drugs, that do not penetrate across a functioning BBB (i.e., in the absence of its significant breakdown in states such as stroke)?

In previous reviews (e.g., Kell et al., 2011, 2013) we have provided a large list of known influx transporters that *might* in fact be exploited, as well as pointing out that no attempt to increase lipophilicity had ever turned a drug that failed to penetrate the BBB into one that did (Pardridge, 2007).

### How does BDII account for the differential uptake into different tissues within an organism (without invoking efflux transport reactions that have not been measured)?

In a similar vein, there is a highly heterogeneous uptake of specific drugs into different tissues, again despite the fact that lipids are not thought to differ substantively between tissues. As above, PBIN has no trouble explaining this in terms of a differential expression of suitable transporters in different tissues—and again a lack of permeability in their absence is expected. For drugs for which efflux transporters in specific tissues are not known, how do proponents of the BDII theory explain the virtually complete lack of uptake of those drugs, including lipophilic drugs, by different tissues or different cells of the same tissue?

**Figure 9:**
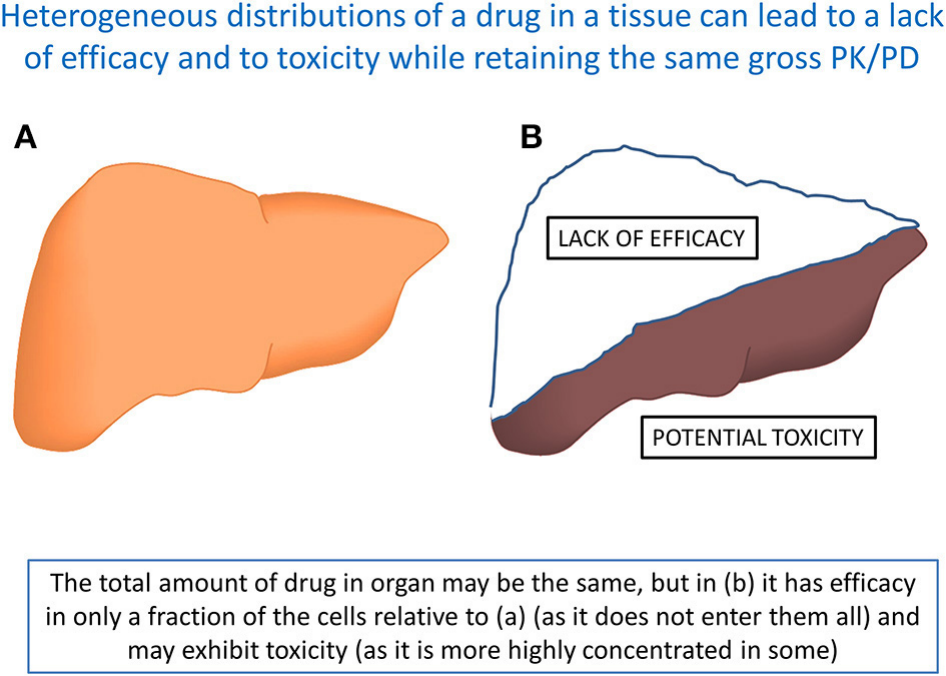
**A set of circumstances in which two otherwise identical organs, that take up the same total amount of a drug and may have indistinguishable PK/PD, nevertheless display entirely different behaviors because of the intercellular heterogeneity**. Organ **(A)** may display favorable efficacy and toxicity profiles, while in organ **(B)** shows both a lack of efficacy (in at least part of the organ) and toxicity (in another part). Note that the total amount of tissue is the same in **(A,B)**. Such phenomena may well underlie the two most common causes of attrition (Cook et al., 2014).

Surprisingly few good data on this are available in the open literature, though in some cases one can see that the variation in concentration of a drug in different tissues (e.g., as measured by tissue:plasma ratio) can be massive (e.g., Miraglia et al., 2010; Oballa et al., 2011; Pagliarusco et al., 2011; Pfefferkorn et al., 2012). Note that when these kinds of measurements are made directly there is a highly heterogeneous distribution of drugs between different cells in the same tissue (e.g., Khatib-Shahidi et al., 2006; Cornett et al., 2008; Nilsson et al., 2010; Römpp et al., 2010, 2011; Castellino et al., 2011; Marko-Varga et al., 2011, 2012; Ait-Belkacem et al., 2012; Shahidi-Latham et al., 2012; El-Mashtoly et al., 2014; Gessel et al., 2014). The PBIN theory explains this straightforwardly in terms of the heterogeneous distribution of transporters, which is both well-known and measurable [see e.g., (http://proteinatlas.org/) (Persson et al., 2006; Pontén et al., 2008)]. A consequence of this highly heterogeneous distribution (Figure 9) is that one can find or predict circumstances in which, while the gross PK/PD of a drug's interactions at the level of an organ may not change, the heterogeneous distribution of a drug that might otherwise be efficacious and non-toxic means that it is simultaneously both non-efficacious and toxic (the two main causes of attrition in drug development; Arrowsmith, 2011; Hann, 2011; Arrowsmith and Miller, 2013; Cook et al., 2014).

### How does BDII account for the differential uptake into the same tissues in different organisms (without invoking efflux transport reactions that have not been measured)?

In a variant of the same question, there are considerable differences in uptake of specific drugs into similar tissues of different organisms (often used as models for human pharmacokinetics and pharmacodynamics), again despite the fact that lipid biophysical properties are not thought to differ substantively between tissues of human and animal models. Again, PBIN has no trouble explaining this in terms of a differential expression of different transporters in different species—and a lack of permeability in their absence is *expected*. For drugs for which efflux transporters in the specific tissues of different species are not known, how do proponents of the BDII theory explain the extremely large variability in uptake of drugs, including lipophilic drugs, that can be observed between the same organs and tissues in different species?

Such data are comparatively unavailable in the academic literature, and we strongly encourage their publication so that people can see the extent of the inter-species variation of drug uptake into particular organs or tissues, which variation can again be considerable (e.g., Shilling et al., 2006; Shitara et al., 2006; Li et al., 2008; Furihata et al., 2010; Chu et al., 2013a; Grime and Paine, 2013; Musther et al., 2014).

## Some further areas where the hypothesis of dominant transporter activity (PBIN) has strong predictive and explanatory power, but where a biophysical view based on bilayer lipoidal diffusion (BDII) does not

The above described a number of areas where the expectations of BDII did not provide easy explanations of observable phenomena (in a way that PBIN could). Another important scientific tenet relates to the idea that theories with predictive power are to be preferred over those that lack useful and novel predictions. Thus, we next mention a number of areas in which the view that drugs hitchhike on transporters as their dominant mode of transmembrane transport (PBIN) makes important predictions that do not follow obviously (or even at all) from the view that most or all of drug transport is by diffusion across lipid bilayers (BDII).

### Drug-metabolite likenesses

Since the influx transporters that are used by pharmaceutical drugs were not selected by natural evolution for these purposes, nor for the benefit of the pharmaceutical industry more generally, they must be there for other reasons. The most obvious “other reasons” are for the transport of small molecule nutrients or intermediary metabolites (and, in at least some cases, the natural substrates are indeed known), and of course the molecular targets of many drugs are proteins that interact with natural metabolites. The recent availability of a consensus reconstruction of the human metabolic network (Swainston et al., 2013; Thiele et al., 2013) means that it is now possible to compare “all” known metabolites with “all” drugs. The principle of molecular similarity (Gasteiger, 2003; Bender and Glen, 2004; Oprea, 2004; Sheridan et al., 2004; Maldonado et al., 2006; Eckert and Bajorath, 2007) indicates that drugs, especially those that are transported, should therefore resemble metabolites to a greater or lesser extent. The full analysis is presented elsewhere (O'Hagan et al., 2014), but, in line with previous indications (Feher and Schmidt, 2003; Karakoc et al., 2006; Gupta and Aires-De-Sousa, 2007; Dobson et al., 2009b; Khanna and Ranganathan, 2009, 2011; Peironcely et al., 2011; Zhang et al., 2011a; Chen et al., 2012; Walters, 2012; Hamdalla et al., 2013), it seems that the chemical structures of drugs do indeed resemble natural human metabolites. It is not obvious that BDII has anything to contribute to this, whereas PBIN has clear and strong predictive power. In particular (O'Hagan et al., 2014), using the MACCS encoding of 166 common substructures (Durant et al., 2002), we find that 90% of all marketed drugs have a Tanimoto similarity (Maggiora et al., 2014) of at least 0.5 to at least one metabolite (and in most cases to many more). “While this does not mean, of course, that a molecule obeying (that) rule is likely to become a marketed drug for humans, it does mean that a molecule that fails to obey the rule is statistically most unlikely to do so” (O'Hagan et al., 2014). This provides a useful filter for candidate drugs, as well as a major incentive to make candidate drugs more metabolite-like.

### Heterogeneous drug distribution as a cause of both unexpected toxicity and lack of efficacy

Nowadays, the two most common causes of attrition in drug development are lack of efficacy and toxicity (Hann, 2011; Cook et al., 2014). The latter is arguably more understandable since (on elementary statistical grounds alone) every individual is biochemically very different from others (Williams, 1956). As the numbers of recipients tested increases during drug development phases 1–3, one is more likely to find individuals that display toxicity as a result of comparatively rare differences in genetic make-up, lifestyle (“environment”), or Gene x Environment interactions. Even a small amount of toxicity may thus be enough to kill off a drug candidate during its development. The former is less easy to understand, however, since if a drug was efficacious in early phases why may it not be later? One possible explanation comes from the heterogeneous distribution of drug transporters. Thus, Figure 9 shows two otherwise identical organs that take up the same total amount of a drug and may thus have indistinguishable PK/PD. In (a) the drug is distributed homogeneously, while in (b) only ca one third of the cells take up the drug [to three times the concentration of that in (a)], while ca two-thirds of the cells take up none. Obviously those cells (hence the tissue) in (b) will suffer from a lack of efficacy, even though the gross PK/PD measured macroscopically (across the organ) appeared normal, while the “unexpected” accumulation of drugs in other cells might well lead to toxicity. The solution to this is to use single-cell analyses (Davey and Kell, 1996) (and see above), because biochemical systems are neither homogeneous nor ergodic (Kell et al., 1991).

### Molecular dynamics simulations

We noted (above) the rather dismissive attitude taken toward computational modeling in some quarters [“The studies cited (Leontiadou et al., 2004, 2007) are computational simulations (so-called molecular dynamics, MD) of Na^+^ and Cl^−^ ion (non-drug-like) transport under unusual conditions” (Smith et al., 2014)]. It is not clear what the “unusual conditions” are supposed to be, but molecular dynamics simulations provide an approach to many experimentally intractable problems that is extremely well-established throughout science and engineering (see above and e.g., Karplus and Kuriyan, 2005; Dror et al., 2012), and indeed the 2013 Nobel Prize in Chemistry was awarded to three of its pioneers − Martin Karplus, Michael Levitt, and Arieh Warshel. Historically, such simulations have been somewhat limited by the discrepancy between the required and available amounts of computational power, but the growth in computer power, improvements in sampling regimes and other aspects of software (and in some cases the development of dedicated hardware, e.g., Shaw et al., 2008; Dror et al., 2011) are opening up *de novo* simulation on unprecedented timescales (e.g., in protein folding; Lindorff-Larsen et al., 2011; Raval et al., 2012; Piana et al., 2013). We predict that very soon it will be possible to provide accurate simulations of phospholipid bilayer membranes that both lack and contain proteins (and lipids) of the correct type and volume fraction (as in Figure 4), and that these will show precisely the molecular pathways that important drug (and other) molecules do and do not use to cross them. We also predict that as experimental systems more closely approximate real biomembranes, the transport occurring via any lipoidal bilayer portion will become increasingly negligible.

### Mass spectrometric imaging of drugs and transporters

As mentioned above, a great many examples now exist of the heterogeneous distribution of drugs in and between tissues as assessed by imaging mass spectrometry (e.g., Khatib-Shahidi et al., 2006; Cornett et al., 2008; Nilsson et al., 2010; Römpp et al., 2010, 2011; Castellino et al., 2011; Marko-Varga et al., 2011, 2012; Ait-Belkacem et al., 2012; Shahidi-Latham et al., 2012; Rumiato et al., 2013; El-Mashtoly et al., 2014; Gessel et al., 2014). We predict that the imaging of both proteins (or signature peptides derived therefrom) and drugs *in the same locations in tissues* will continue to be a powerful strategy for assessing which drugs are taken up by which transporters. It is not obvious that BDII can predict any such thing such as a relationship between particular lipids and particular transmembrane drug uptake (in an external validation set).

### Adverse drug reactions

As well as their immense therapeutic benefits, pharmaceutical drugs can have unwelcome effects on those who take them, another huge topic usually referred to as “adverse drug reactions” (ADRs). It is a massively important issue (e.g., Uetrecht, 2010), accounting for more than 5% of UK hospital admissions (Pirmohamed et al., 2004; Davies et al., 2007; Kongkaew et al., 2008) and even more adverse events *after* hospital admission (Clavenna and Bonati, 2009; Davies et al., 2009; Miguel et al., 2012; Smyth et al., 2012) (and these are probably underestimates; Hazell and Shakir, 2006). Most are avoidable (Pirmohamed et al., 2004; Smyth et al., 2012), and considerable pharmacogenetic, and pharmacogenomics evidence reflects the roles of drug transporters in ADRs (Meyer, 2000; Nakamura, 2008; Ward, 2008; Pirmohamed, 2010, 2012, 2014; Tohkin et al., 2010; Uetrecht, 2010; Clarke and Cherrington, 2012; Daly, 2012, 2013; Giacomini et al., 2012; Wei et al., 2012; Stankov et al., 2013; Yip et al., 2014), again reflecting their considerable significance relative to any bilayer lipoidal diffusion (where again it is not obvious how BDII has anything of substance to say).

### Transporter pharmacogenomics

A further prediction that follows from the recognition of the widespread use of transporters by drugs (but not from models of bilayer lipoidal diffusion) is that one ought to be able to detect these transporters via the effects of genetic mutations (i.e., polymorphisms) on transport activity (they may either increase or decrease transport activity for a given substrate) (e.g., Ishikawa et al., 2004, 2013a,b; Bosch, 2008; Errasti-Murugarren and Pastor-Anglada, 2010; Franke et al., 2010; Lee, 2010; Nies and Schwab, 2010; Sissung et al., 2010, 2012; Aw et al., 2011; Li and Bluth, 2011; Pirmohamed, 2011, 2013, 2014; Stieger and Meier, 2011; Yonezawa and Inui, 2011; Kiyotani et al., 2012; Lai et al., 2012; Saadatmand et al., 2012; Wei et al., 2012; Giacomini et al., 2013; Yiannakopoulou, 2013). One well-known example, based on genome-wide association studies, is the effect of a particular SNP in *SLCO1B1*, previously known as OATP1B1, a bile acid and statin transporter (Hagenbuch and Meier, 2004; Hagenbuch and Stieger, 2013), on the myopathy that can be induced by particular statins (Link et al., 2008; Becquemont, 2009; Voora et al., 2009; Amur et al., 2010; Fahrmayr et al., 2010; Donnelly et al., 2011; Giorgi et al., 2011; Maggo et al., 2011; Nakanishi and Tamai, 2012; Wilke et al., 2012; Carr et al., 2013; Giacomini et al., 2013; Shitara et al., 2013; Yiannakopoulou, 2013; Ramsey et al., 2014; Rose et al., 2014; Tsamandouras et al., 2014).

### Transporter-mediated drug-drug interactions

Yet another area for which the transporter-mediated route gives straightforward understanding and predictions (whereas the bilayer lipoidal diffusion mechanism has little to say) is in the area of transporter-mediated drug-drug interactions (DDI) (and indeed food-drug interactions). An elementary consequence of standard enzyme kinetics is that molecules using the same protein may compete with or inhibit each other, in this case each other's transport. This is a simply vast topic, so (notwithstanding earlier critiques of summarizing via the enormous review literature), we here simply point out several useful and recent reviews (from the last 3 years only) that describe in detail the many named and genetically identified transporters that are involved in DDI (Han, 2011; Kido et al., 2011; Klatt et al., 2011; König, 2011; Maeda et al., 2011; Marzolini et al., 2011; Müller and Fromm, 2011; Riches et al., 2011; Shitara, 2011; Zhang et al., 2011b; Bi et al., 2012; Elsby et al., 2012; Feng et al., 2012, 2013, 2014; Fromm, 2012; Grandvuinet et al., 2012; Karlgren et al., 2012; Keogh, 2012; Lepist and Ray, 2012; Nies et al., 2012; Sissung et al., 2012; Sprowl and Sparreboom, 2012, 2014; Takanohashi et al., 2012; Varma et al., 2012; Yeo et al., 2012, 2013; Yoshida et al., 2012, 2013; Kis et al., 2013; König et al., 2013; Maeda and Sugiyama, 2013; Sugiyama and Steffansen, 2013; Tang et al., 2013; Zamek-Gliszczynski et al., 2013; Goswami et al., 2014; Tannenbaum and Sheehan, 2014; Vildhede et al., 2014). We are not aware of any papers that showed such DDI based on any measured competition for transport via the phospholipid bilayer.

### Toward targeted therapeutics

Knowledge of transporters and their heterogeneous selectivities and distributions can be used to target particular drugs to particular tissues (Dobson and Kell, 2008). Thus, “In recent years, drug discovery researchers have also utilized knowledge about transporter uptake to enhance drug exposure to certain tissues. For example, liver specific transporters (OATP1B1 and 1B3) selectively increase liver concentration of their substrates, which minimize the exposure to peripheral tissue and reduce toxicity (Oballa et al., 2011; Pfefferkorn et al., 2012)” (Smith et al., 2014), though we also note that this can lead to toxicities in particular cases, e.g., Zhang et al. (2013).

**Figure 10:**
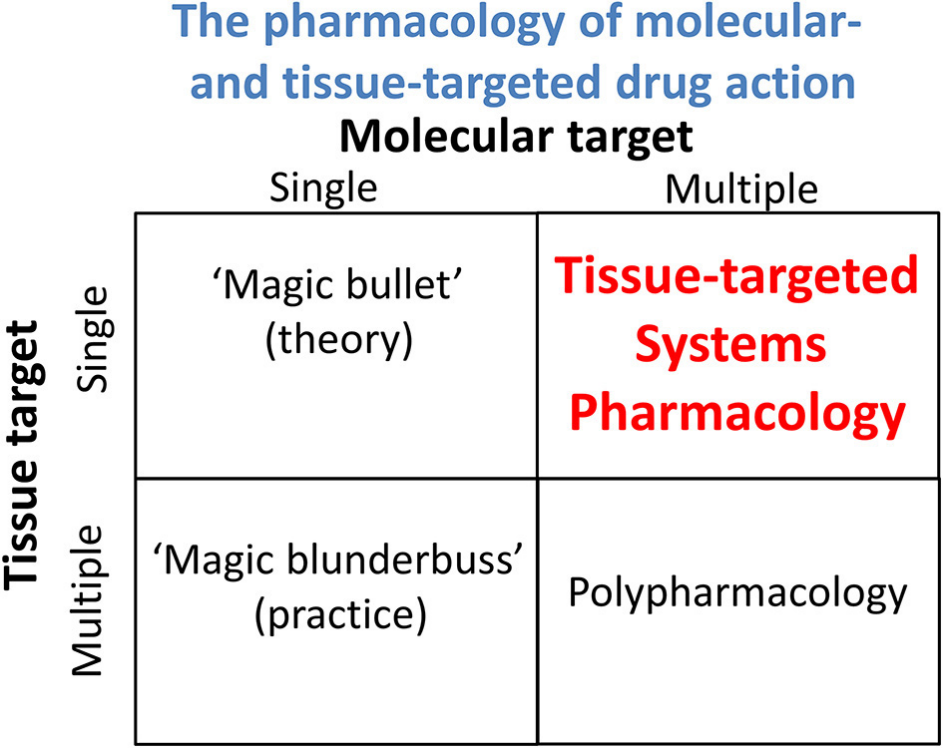
**A “Boston matrix” comparing the activities of drugs in terms of whether their molecular and/or their tissue targets are each either single or multiple**.

Indeed, one can devise a 2 × 2 matrix of *molecular* vs. *tissue* targeting in drug development (Figure 10); however, its most important quadrant is presently missing (see Figure 10). Thus, the “magic bullet” is a phrase that was coined by Paul Ehrlich (Bosch and Rosich, 2008), initially with regard to anti-infectives, to describe a chemical that specifically inhibits a disease-causing target. Much of modern pharmacology relies precisely upon this principle, and many highly potent drugs have been developed (Strebhardt and Ullrich, 2008). However, most effective drugs are, in fact, active on *multiple* targets (e.g., Hopkins, 2008; Mestres and Gregori-Puigjané, 2009; Kell et al., 2013) and there is an increasing recognition (e.g., Boran and Iyengar, 2010; Metz and Hajduk, 2010; Xie et al., 2012; Jalencas and Mestres, 2013; Medina-Franco et al., 2013; Peters, 2013; Anighoro et al., 2014) as we move toward a network or systems pharmacology (e.g., Hopkins, 2008; Van Der Graaf and Benson, 2011; Cucurull-Sanchez et al., 2012; Rostami-Hodjegan, 2012; Waldman and Terzic, 2012; Bai and Abernethy, 2013; Csermely et al., 2013; Jenkins and Ma'ayan, 2013; Kell and Goodacre, 2014) that *polypharmacology* (one drug, multiple targets) is actually a desirable goal. While this can be achieved with combination therapies (multiple drugs, multiple targets) (e.g., Borisy et al., 2003; Zimmermann et al., 2007; Lehár et al., 2008), an advantage of polypharmacology is that the pharmacokinetics of a single agent are considerably simpler. However, with regard to *tissue targeting*, the “magic bullet” is more like a “magic blunderbuss” in that the drug can, in principle, bind to its targets *in whichever tissue they are found* so that it is largely unselective, and this is also true for agents exhibiting polypharmacology. This is obviously particularly undesirable for cytotoxic drugs such as anti-cancer agents, and leads to many examples of unwanted toxicity. By including tissue targeting as an explicit goal, much lower amounts of active drug can be given, thereby improving their therapeutic index massively. The starting point for achieving this is indeed the recognition that drugs enter cells more or less solely by hitchhiking on transporters normally involved in the transport of intermediary metabolites, rather than by diffusing indiscriminately through any and every phospholipid bilayer. A striking example comes from the work of Pfefferkorn and colleagues, who noted a value of 250,000 for the hepatocyte:myocyte ratio of a particular transporter-targeted drug (Pfefferkorn et al., 2011). It is not obvious how any view of a significant bilayer transport occurring (as in BDII) can sensibly account for this without *ad hoc* extra hypotheses, whereas the view that any “background” lipoidal bilayer transport is negligible finds it very easy to do so.

Thus, this possibility of cell or tissue targeting is a very clear prediction from PBIN that we consider has considerable utility in improving the potential therapeutic windows of active drugs, and highlights the need to develop pharmacophores for the more important transporters (Gleeson et al., 2011). As with all the other predictions and postdictions of PBIN, the predicted potential for selective, transporter-mediated targeting is amply fulfilled (e.g., Friend and Pangburn, 1987; Erion, 2007; Oballa et al., 2011; Pfefferkorn et al., 2011; Powell et al., 2011; Ramtohul et al., 2011; Reiling et al., 2011; Brunschweiger and Hall, 2012; Lachance et al., 2012a,b; Birsoy et al., 2013; Filipski et al., 2013; Liu, 2013; Pfefferkorn, 2013; Stevens et al., 2013; Tu et al., 2013; Sun et al., 2014; Tsume et al., 2014).

## Concluding remarks

There is considerable value in having an intellectual debate in this space; “accurate understanding of drug permeation mechanisms is important for drug development success” (Smith et al., 2014). Here, rather than being entirely fundamentalist (“When one admits that nothing is certain one must, I think, also admit that some things are much more nearly certain than others”; Russell, 1947), we have chosen to rehearse the common Popperian view of science as a scientific principle that can help to discriminate the virtues of the two competing hypotheses that we term BDII and PBIN. In laboratory and experimental science, this means varying parameters (often referred to as “independent variables” or causes) and observing their effects. This contrasts completely with the many observations, widely cited in support of BDII, of the mere covariation of two dependent variables.

While we think that the case is strongly made for the far greater utility and explanatory power of PBIN, we trust that this analysis will help readers of this journal to draw their own conclusions and to design better experiments to assist the modern drug discovery process. Hopefully this will also help us overcome the problem of what have been called the “‘unknown knowns’; these are those things that are known but have become unknown, either because we have never learnt them, or forgotten about them, or more dangerously chosen to ignore” (Hann, 2011).

### Conflict of interest statement

The authors declare that the research was conducted in the absence of any commercial or financial relationships that could be construed as a potential conflict of interest.
